# Pan-cancer characterization of metabolism-related biomarkers identifies potential therapeutic targets

**DOI:** 10.1186/s12967-021-02889-0

**Published:** 2021-05-24

**Authors:** Guoshu Bi, Yunyi Bian, Jiaqi Liang, Jiacheng Yin, Runmei Li, Mengnan Zhao, Yiwei Huang, Tao Lu, Cheng Zhan, Hong Fan, Qun Wang

**Affiliations:** 1 Department of Thoracic Surgery, Zhongshan Hospital, Fudan University, No. 180 Fenglin Rd, Xuhui District, Shanghai, 200032 China; 2 Department of Biostatistics, Public Health, Fudan University, Shanghai, 200000 China

**Keywords:** Metabolism, Glycolysis, Pan-cancer, Oxidative phosphorylation, Warburg effect

## Abstract

**Background:**

Generally, cancer cells undergo metabolic reprogramming to adapt to energetic and biosynthetic requirements that support their uncontrolled proliferation. However, the mutual relationship between two critical metabolic pathways, glycolysis and oxidative phosphorylation (OXPHOS), remains poorly defined.

**Methods:**

We developed a “double-score” system to quantify glycolysis and OXPHOS in 9668 patients across 33 tumor types from The Cancer Genome Atlas and classified them into four metabolic subtypes. Multi-omics bioinformatical analyses was conducted to detect metabolism-related molecular features.

**Results:**

Compared with patients with low glycolysis and high OXPHOS (LGHO), those with high glycolysis and low OXPHOS (HGLO) were consistently associated with worse prognosis. We identified common dysregulated molecular features between different metabolic subgroups across multiple cancers, including gene, miRNA, transcription factor, methylation, and somatic alteration, as well as investigated their mutual interfering relationships.

**Conclusion:**

Overall, this work provides a comprehensive atlas of metabolic heterogeneity on a pan-cancer scale and identified several potential drivers of metabolic rewiring, suggesting corresponding prognostic and therapeutic utility.

**Supplementary Information:**

The online version contains supplementary material available at 10.1186/s12967-021-02889-0.

## Background

Metabolic reprogramming improves cancer cell’s fitness to provide a selective advantage during tumorigenesis [1]. The key characteristic of tumor metabolism is the phenomenon that instead of oxidative phosphorylation (OXPHOS), cancer cells prefer to enhance glucose uptake and rely on aerobic glycolysis, even in the presence of oxygen and fully functioning mitochondria, also known as Warburg effect [2–4]. Pyruvate, an important metabolite in glycolytic process, is more likely to be transferred to lactate in cancer cells rather than enter tricarboxylic acid cycle followed by OXPHOS. This metabolic rewiring has been proposed to be an adaption mechanism to support the energetic and biosynthetic requirements of cellular processes and uncontrolled proliferation, as well as the acidification of microenvironment for enhanced invasiveness [4–6]. The “branching point” for glycolysis and OXPHOS lies in how and where the conversion of pyruvate occurs, which is regulated by multiple important oncogenic factors and tumor suppressor pathways. However, this notion was sharply challenged recently. For example, Xian et al.’s study on tumor metabolic microenvironment at single-cell resolution noted the significant positive correlation between OXPHOS and glycolysis in melanoma and HNSC [7]. Meanwhile, a study in mice bearing KRAS-driver tumors shows that non-small cell lung tumors demonstrated higher levels of both glycolysis and OXPHOS compared with adjacent normal tissues [8]. Given the existed controversy on this issue, it is worthy to reconsider the balance between these two metabolic pathways.

Previous researchers have reported considerable metabolic heterogeneity both within and among cancer types. However, a comprehensive study is still warranted to illustrate the mutual relationship between glycolysis and OXPHOS, as well as its regulatory mechanism and clinical implication. Herein, by employing multi-omics molecular data from The Cancer Genome Atlas (TCGA) across 33 cancer types, we stratified patients into different subgroups based on the expression patterns of glycolytic and OXPHOS genes, and explore their survival status and key-regulators potentially driving metabolic alteration from multiple dimensions.

## Methods and materials

### TCGA data acquisition and preprocessing

Level 4 gene sequencing (FPKM normalized), mature microRNA (miRNA) expression, DNA methylation, somatic single nucleotide variants (SNVs) and somatic copy number variation (CNVs), and corresponding clinical data of 33 human cancers in TCGA were downloaded from the UCSC Xena browser (GDC hub: https://gdc.xenahubs.net). We removed patients whose clinical outcome information, including survival time and vital status, were vague or absent. We also downloaded mass proteomic spectrum data for breast invasive carcinoma (BRCA) and ovarian serous cystadenocarcinoma (OV) from the Clinical Proteomic Tumor Analysis Consortium (CTPAC) [9, 10].

### Pan-cancer metabolic scoring and subtype classification

Genes sets downloaded from MSigDB [https://www.gsea-msigdb.org/gsea/msigdb] and Reactome [https://reactome.org/] were used as candidate glycolytic and OXPHOS signature genes, respectively. Consensus clustering was performed using *ConsensusClusterPlus* package [11] (Parameters: reps = 1000, pItem = 0.8, pFeature = 1; clustering algorithm: K-means; distance metric: Euclidean distances), thus the signature genes that exhibited similar expression mode for glycolysis and OXPHOS pathway were identified. Based on the two “filtered” gene signatures, the Glycolysis score and OXPHOS score were respectively computed from RNA sequencing of each bulk sample using the gene set variation analysis (GSVA) algorithm in the *GSVA* package [12], an unsupervised gene set enrichment method that computes an enrichment score by integrating the collective expression of a given gene set relative to the other genes in the sample. It has been reported that GSVA outperforms single-cell gene set enrichment analysis (ssGSEA) when measuring the signal-to-noise ratio in differential gene expression and differential pathway activity identification analyses because GSVA includes normalization of gene expression aimed at reducing the noise of the data [13]. Besides, we assessed the correlation of Glycolysis and OXPHOS scores across all five “primary” signatures mentioned above (4 for glycolysis and 1 for OXPHOS). For each pair of signatures, the Pearson’s correlation was calculated on a pan-cancer scale. Afterward, in each cancer type, the medians for the Glycolysis score and OXPHOS score were used to assign the patients enrolled in this study into different metabolic subgroups. Survival analysis based on log-rank test (p < 0.05) and univariable Cox proportional hazard model regression analysis was performed using *survival* and *survminer* packages, and corresponding Kaplan–Meier plots were produced by *ggplot2* package. The results of survival analyses are presented as hazard ratios (HR) with corresponding 95% confidence intervals (95%CI).

### Analysis of metabolite profiling data

We obtained metabolite profiling data and corresponding RNA sequencing data on 48 patients with breast cancer [14]. We focused on the metabolites belonging to glycolytic and OXPHOS pathways. For each metabolite, we computed the Pearson’s correlation between its abundance and the expression levels of genes in corresponding metabolic pathways. Significance of hypothesis testing was adjusted with false discovery rates (FDR). The correlations with an FDR less than 0.05 were considered to be significant. Next, to assess whether the metabolic pathway signature genes are more informative about actual metabolic activities than other genes, we performed a simulation analysis by randomly selecting a gene set with the same size of the glycolysis pathway and identified the number of genes significantly correlated with the abundance for glycolysis-related metabolites in the same way as the genes belonging to glycolysis signature. This analysis was repeated for 1000 times to generate the background distribution of significant hits from which we evaluate whether the actual observed numbers were significantly higher than random expectation.

### Differential expression and functional enrichment analysis

Differentially expressed genes (DEGs) and miRNAs were identified between different metabolic subgroups across cancer types using the package *limma*, which implements the Benjamini and Hochberg method to estimate gene expression changes using the moderated *t*-test and adjust the P-value as FDR [15]. |Log (fold change) (Log FC)|> 0.5 and adjusted P-value < 0.05 were considered cutoff criteria to screen for differential expression. We combined the results from MiRWalk [http://mirwalk.umm.uni-heidelberg.de/search_mirnas/] and TargetScan [http://www.targetscan.org/] to identify potential targeting relationships between DEGs and miRNAs [16].

For DNA methylation, we employed the TCGA methylation data obtained by the Illumina Human Methylation450 BeadChip array, which contains 485,577 probes (396,066 after filtering invalid probes) covering 99% of RefSeq genes. The methylation levels of each probe were quantified as β-values, which are the ratios of the intensities of methylated and unmethylated alleles. 5′-C-phosphate-G-3′ (CpG) methylation data between different metabolic groups were normalized and compared with the CHAMP pipeline [17]. The algorithm used for differentially methylated CpG sites is similar to that used in the DEG analysis, and the threshold was set as adjusted p-value < 0.05 and an absolute ∆β-value > 0.2. A gene was considered to be differentially methylated if there was at least one differentially methylated CpG in its promoter region.

To identify transcription factor (TF) potentially regulating tumor metabolic status, we adopted previously proposed tumor context-specific TF regulator networks for 21 tumor types in TCGA from package *arcane.network* [18]. Then, given the existing networks, we used the Master Regulator Inference Algorithm (MARINa, from *ssmarina* package) in each cancer type to computationally infer the TF protein activity using the expression profile data of genes that are most directly regulated by a given target as an accurate reporter of its activity. Furthermore, to address the confounding shadow effect, that a regulator may appear to be significantly activated simply because several of its targets may also be regulated by other activated TF (shadow effect) [19, 20], we perform a post-hoc shadow analysis to correct the results from MARINa. Therefore, the potential master TFs passing the shadow analysis with FDR < 0.05 were identified in each cancer type. TFs processing regulatory functions in ≥ 6 tumor types were considered as “common TF,” and the target genes of them were identified using the *ledge* function of *ssmarina* package. Next, the detected target genes, which were regulated by ≥ 3 TFs in at least ten cancer types, were defined as “common target”. The differential expression between different metabolic subgroups of these common TFs and targets in each cancer was computed, and the pan-cancer TF regulatory network was visualized by the use of *circlize* package.

Functional enrichment analyses of the detected DEGs and common TFs were performed with the *clusterProfiler* package [21]. Gene Ontology (GO) terms, including molecular function, biological pathway, and cellular component, were identified with a strict cutoff of adjusted P < 0.05.

### Somatic alteration association analysis

To identify oncogenic somatic alterations that potentially drive metabolic reprogramming, we analyzed the associations of SNVs and CNVs with metabolic subtypes in each cancer type. All results in this section were generated with *maftools* package. We performed Fisher-exact test to determine the association between the metabolic subtypes and a specific differentially mutated gene status, where P-values < 0.05 were considered statistically significant.

Driver CNV associations were assessed within all 33 tumor types for 112 previously described oncogenes and tumor-suppressor genes [22]. For each oncogene, differences in Glycolysis score and OXPHOS score were computed between tumors with copy-number amplification and those without (Wilcoxon test, FDR < 0.05). For each tumor-suppressor gene, differences in Glycolysis score and OXPHOS score were computed between tumors with copy-number loss and those without (Wilcoxon test, FDR < 0.05). The CNV fraction data, also known as the percentage of the genome altered by CNV, were downloaded from cBioportal [https://www.cbioportal.org/], and the correlations between this index and metabolic scores were calculated.

### Pathway functional enrichment analysis

We computed the GSVA score of 57 biological pathways, including 50 hallmark gene sets proposed by Liberzon et al. [23], seven metabolic super-pathways from the latest Reactome annotations [24] in each cancer type, as well as the tumor stemness index [25]. These gene signatures were classified into ten subgroups according to their function: cellular component, development, DNA damage, hallmark metabolism, immune, metabolic products, pathway, proliferation, signaling, and others. The correlations between our metabolic scores and these pathway scores were calculated in each cancer type, respectively (FDR < 0.05).

### Analysis of drug sensitivity data

We downloaded cancer cell line drug sensitivity databases from the Genomics of Drug Sensitivity in Cancer (GDSC; available at https://www.cancerrxgene.org/downloads/anova) [26–28], which includes gene expression data obtained using an Affymetrix HT HG U133A array and drug sensitivity data, presented as the area under the dose–response curve (AUC) values and IC_50_ values (half maximal inhibitory concentration) based on cell viability assays. Using the same classification pipeline, we classified them into metabolic subgroups mentioned above and performed Student’s t-test to determine whether AUC values showed a significant difference among different metabolic subtypes. We also employed *networkD3* package to exhibit the correlations among the drug sensitivity data and metabolism-related DEGs.

## Result

### Dual analysis of glycolytic and OXPHOS gene expression describes the metabolic landscape of cancer

**Fig. 1:**
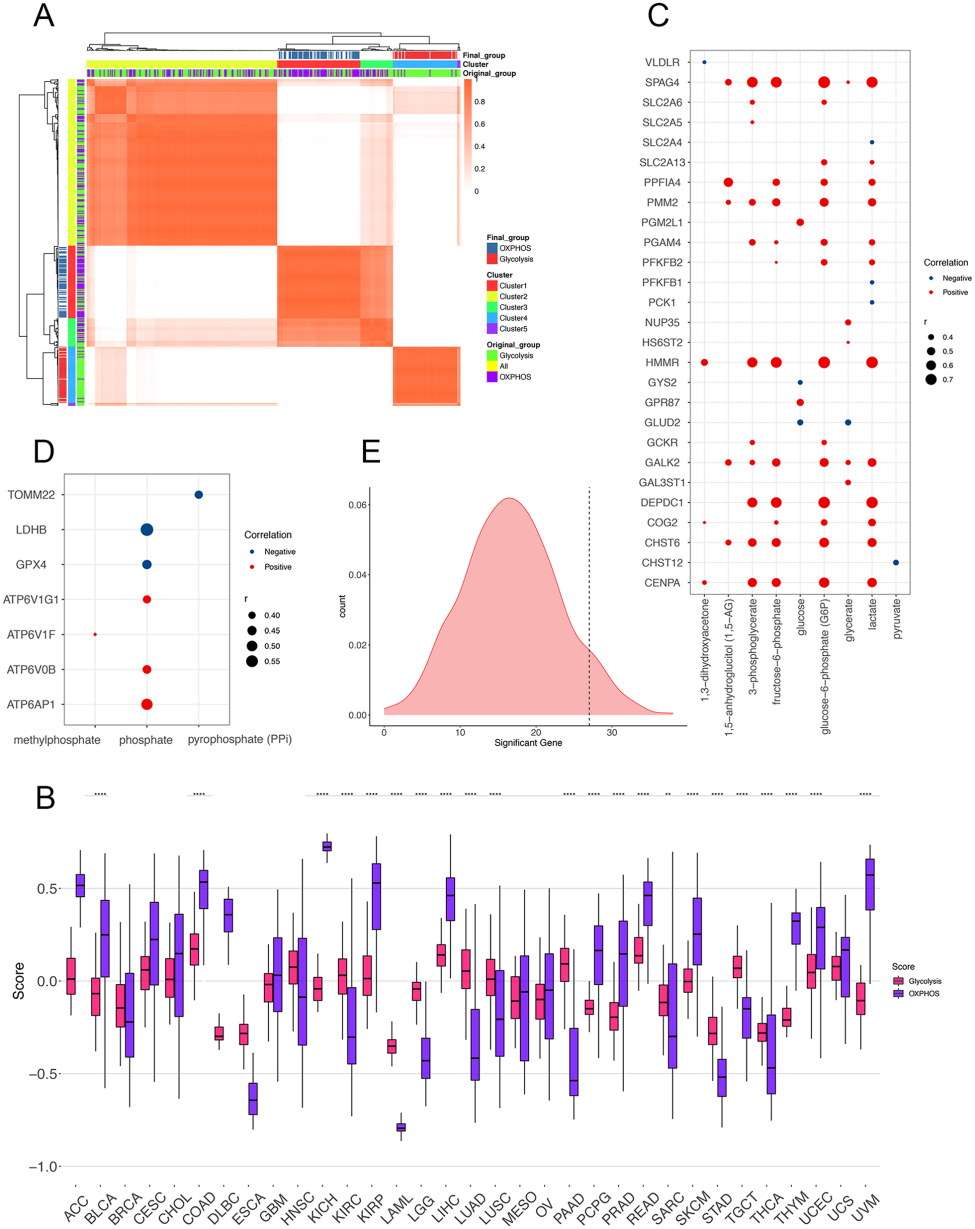
**A** The identification of gene signatures for OXPHOS by consensus clustering algorithm on a pan-cancer scale (k = 5). **B** The distribution of the Glycolysis score and OXPHOS score across 33 cancer types. Within each group, the scattered dots represent the two scores of each patient. The lines in the boxes represent the median value. The bottom and top of the boxes are the 25th and 75th percentiles (interquartile range). The whiskers encompass 1.5 times the interquartile range. The statistical difference of the two scores was compared through the Wilcoxon test. *p < 0.05; **p < 0.01; ***p < 0.001; ****p < 0.0001. **C**, **D** The bubble plot shows the correlations between the expression level of signature genes and the abundance of metabolites annotated to Glycolysis (**C**) and OXPHOS (**D**) pathways. Significant correlations (Pearson’s correlation, FDR < 0.05) are displayed. Red dots indicate a positive correlation, while blue ones for negative. Dot size is proportional to the strength of the correlation. **E** The density plot displays the “background distribution” of significant hits when assessing the correlation between the abundance of glycolysis-related metabolites and expression of random-selected gene sets (n = 72 for each gene set, repeated for 1000 times). The black dashed line indicates the true number. FDR, false discovery rates. *ACC* adrenocortical carcinoma, *BLCA* bladder urothelial carcinoma, *BRCA* breast invasive carcinoma, *CESC* cervical squamous cell carcinoma and endocervical adenocarcinoma, *CHOL* cholangiocarcinoma, *COAD* colon adenocarcinoma, *DLBC* lymphoid neoplasm diffuse large B-cell lymphoma, *ESCA* esophageal carcinoma, *GBM* glioblastoma multiforme, *HNSC* head and neck squamous cell carcinoma, *KICH* kidney chromophobe, *KIRC* kidney renal clear cell carcinoma, *KIRP* kidney renal papillary cell carcinoma, *LAML* acute myeloid leukemia, *LGG* brain lower grade glioma, *LIHC* liver hepatocellular carcinoma, *LUAD* lung adenocarcinoma, *LUSC* lung squamous cell carcinoma, *MESO* mesothelioma, *OV* ovarian serous cystadenocarcinoma, *PAAD* pancreatic adenocarcinoma, *PCPG* pheochromocytoma and paraganglioma, *PRAD* prostate adenocarcinoma, *READ* rectum adenocarcinoma, *SARC* sarcoma, *SKCM* skin cutaneous melanoma, *STAD* stomach adenocarcinoma, *TGCT* testicular germ cell tumors, *THCA* thyroid carcinoma, *THYM* thymoma, *UCEC* uterine corpus endometrial carcinoma, *UCS* uterine carcinosarcoma, *UVM* uveal melanoma

The study design is summarized in Additional file 1: Fig. S1. In order to investigate the landscape of tumor metabolism, we complied a pan-cancer cohort of 9668 cases from 33 cancer types from TCGA and stratified them based on their relative expression levels of glycolytic and OXPHOS pathway genes. Gene signatures representing all enzymes and isoenzymes involved in these two pathways proposed by different studies [23, 24] (Glycolysis = 292; OXPHOS = 200) were integrated together for further analysis to achieve the best comprehensiveness and representativeness when measuring the actual status of the metabolic pathways. However, previous studies have figured out the potential heterogeneity in metabolic gene expression, including the isoenzymes within specific pathways between different cancer types [29–31]. To avoid this problem, we performed consensus clustering to identify the signature genes exhibiting similar expression pattern among multiple tumor types. As shown in Fig. 1A, based on the pan-cancer RNA-sequencing data, the expression level of the genes involved in the signatures mentioned above [23, 24] were categorized into five clusters, in which the cluster 1 (n = 72) mainly consists of glycolytic genes whereas cluster 4 (n = 66) mainly consists of OXPHOS genes. Therefore, we selected these co-expressed genes belonging to the two groups as the glycolysis and OXPHOS pathway gene signatures respectively to be used for describing the status of the two pathways (Fig. 1A, Additional file 9: Data 1). The Glycolysis and OXPHOS scores were then calculated by GSVA based on the corresponding “co-expressed” gene signatures for each cancer patient enrolled in this study (Additional file 10: Data 2). The robustness and representativeness of the two “filtered” metabolic scores could be verified by their strong correlations with the GSVA scores for the above mentioned 2 “primary” signatures on a pan-cancer scale (Additional file 2: Fig. S2A, Glycolysis: r = 0.75; OXPHOS: r = 0.96). However, the correlation between these two scores is not completely opposite to each other as expected (r = 0.33), indicating the complicated mutual relationship between the two pathways.

The Glycolysis and OXPHOS scores exhibited distinct distribution patterns in different cancer types (Fig. 1B), highlighting the metabolic diversity among tumor types. For example, in terms of Glycolysis score, the median (interquartile range) was − 0.30 (− 0.33 to − 0.24) for lymphoid neoplasm diffuse large B-cell lymphoma (DLBC); for acute myeloid leukemia (LAML), the value was even lower: − 0.35 (− 0.39 to − 0.31). These were in contrast to the solid tumor types such as colon adenocarcinoma (COAD): 0.17 (0.08–0.27), liver hepatocellular carcinoma (LIHC): 0.014 (0.08–0.21), and rectum adenocarcinoma (READ): 0.14 (0.07–0.24). These results suggest that glycolysis-related genes are significantly upregulated in digestive malignancies such as COAD, LIHC, and READ, thus leading to the increased score, while the Glycolysis scores are much lower in non-solid tumors like DLBC and LAML. As for OXPHOS score, we observed higher viability among different tumor types (Fig. 1B). LAML also exhibits lowest score: − 0.79 (− 0.81 to − 0.77), whereas kidney chromophobe (KICH) has the highest: 0.72 (0.69–0.75).

We further adopted metabolite data to investigated whether the expression patterns of metabolic pathway genes and corresponding scores could reflect actual metabolic activities in tumor patients. Considering the unavailability of metabolite data in TCGA, we enrolled an independent dataset that provides parallel gene expression and metabolites profiling data and focused on the 12 metabolites that had been annotated to Glycolysis (n = 9) and OXPHOS (n = 3) pathways [14]. The correlations between metabolite abundance and corresponding pathway genes were calculated and exhibited in Fig. 1C, D (only those with an FDR < 0.15). For example, HMMR, a hyaluronan associated gene from the Glycolysis signature, was found to be significantly correlated with 5 glycolytic metabolites, with 4 of them displaying correlation > 0.6 (3-phosphoglycerate, r = 0.63, FDR < 0.001; fructose-6-phosphate, r = 0.68, FDR < 0.001; lactate, r = 0.75, FDR < 0.001; glucose-6-phosphate (G6P), r = 0.76, FDR < 0.001). And we also noticed SPAG4, whose expression level was significantly in agreement with six glycolytic metabolites (Fig. 1C). As for OXPHOS pathway, only three metabolites were annotated, but we still detected the significant correlation between phosphate, a key factor in OXPHOS process, and five genes in this pathway (Fig. 1D). However, the correlation between pathway scores and metabolites are quite weak (Additional file 2: Fig. S2B–E). This result might be due to the integrative nature of the Glycolysis and OXPHOS score, which represent the overall activity of the corresponding metabolic process rather than the abundance of a specific metabolite. Besides, to assess the statistical significance of the number of significant hits detected, we conducted a simulation analysis to compare the number of genes with significant signals from Glycolysis signature with those based on random gene sets of the same size (n = 72, FDR < 0.05). Strikingly, the mean number of genes exhibiting significant correlations with glycolytic metabolites in 1000 random samples was only 16.862, whereas the real number of significant genes in glycolytic signatures was 27 (Student t-test: p < 0.001, Fig. 1E). These results demonstrate that the expression patterns of metabolic pathway genes do reflect actual metabolic activities.

### Classification of metabolic subtypes and their prognostic value

**Fig. 2:**
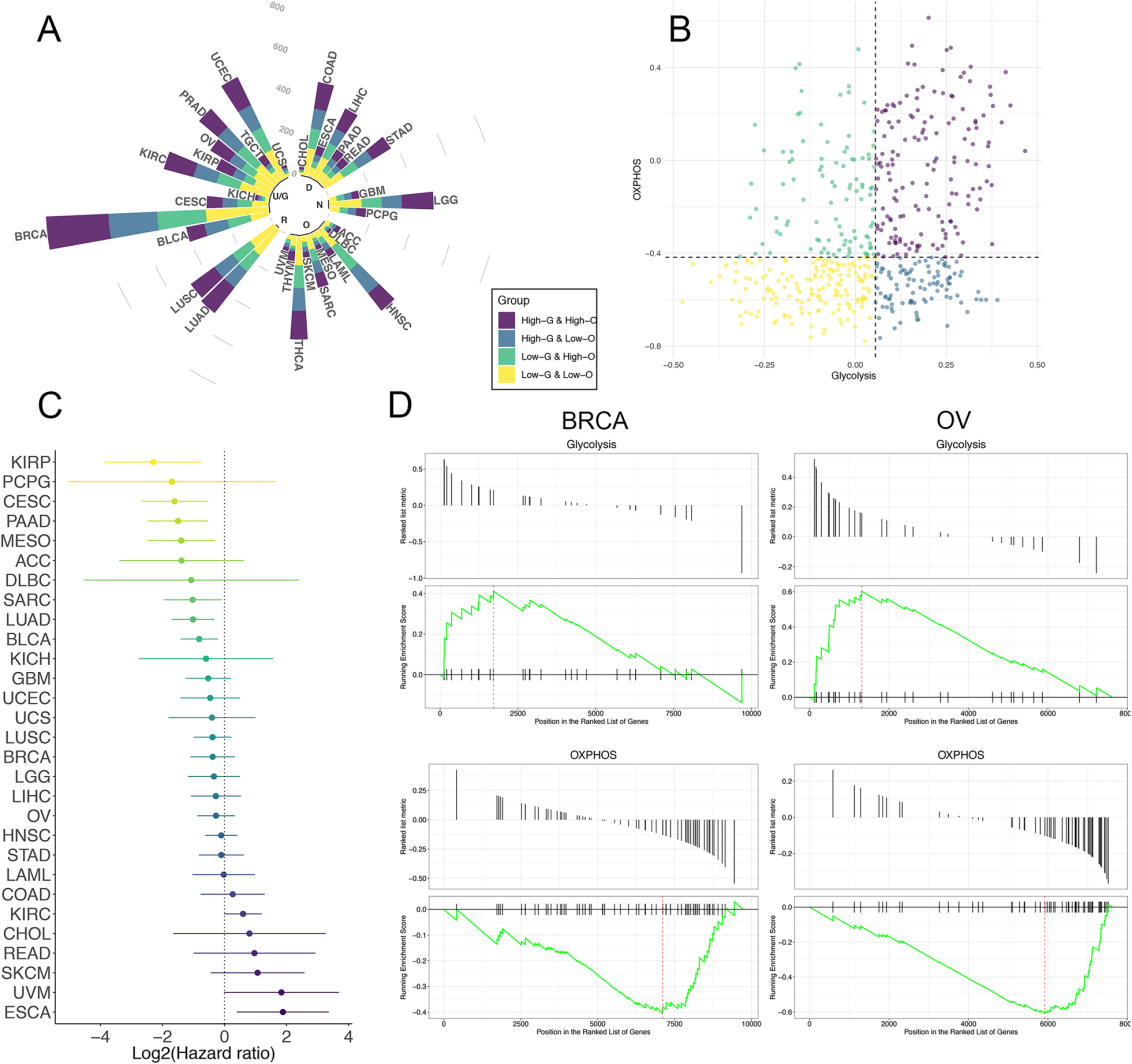
**A** The circular bar plot displays the percentages and numbers of samples in four different metabolic subgroups across multiple cancer types. The cancers are also annotated with the physiology systems they belong to: ‘R’, Respiratory system; ‘U/G’, Urinary system and Genital system; ‘D’, Digestive system; ‘N’, Nervous system; ‘O’, Other systems. **B** The scatter plot showing the distribution of Glycolysis score (x-axis) and OXPHOS score (y-axis) in tumor patients. Here we took lung adenocarcinoma as an example to illustrate the rule for subgroup classification: patients were assigned to four metabolic subgroups according to the median value of the two scores. **C** The prognostic value of metabolic classification (LGHO vs HGLO) in each cancer type, shown in the forest plot with corresponding hazard ratio (HR, log-transformed) and 95% confident interval (95%CI, log-transformed). **D** Gene set enrichment analysis (GSEA) based on mass proteomic spectrum data for BRCA and OV indicated that the proteins in the Glycolysis signature were significantly enriched in HGLO group, while proteins in OXPHOS signature were enriched in LGHO group

We classified all the cancer patients enrolled in this study into four metabolic subgroups according to their Glycolysis and OXPHOS scores: High-glycolysis and high OXPHOS (HGHO), High-glycolysis and low OXPHOS (HGLO), low-glycolysis and high OXPHOS (LGHO), and Low-Glycolysis & Low OXPHOS (LGLO) (Fig. 2A, Additional file 10: Data 2). The medians for the two scores in each cancer type were considered as the cut-off value for the definition of “High” and “Low” (Using LUAD as an example, Fig. 2B).

We next investigated the clinical relevance of the metabolic subtypes mentioned above, since overall survival serves as a key index for evaluating tumor aggressiveness (Fig. 2C, Additional file 3: Fig. S3). Notably, compared with HGLO, LGHO subtypes were consistently associated with better prognosis in 23 out of 33 tumor types, with 7 exhibiting statistical significance, including cervical squamous cell carcinoma and endocervical adenocarcinoma (CESC; HR: 0.33 (0.16–0.70), p = 0.003), kidney renal papillary cell carcinoma (KIRP; HR: 0.20 (0.07–0.60), p = 0.004), mesothelioma (MESO; HR: 0.38 (0.18–0.80), p = 0.011), pancreatic adenocarcinoma (PAAD; HR: 0.35 (0.18–0.69), p = 0.002), and LUAD (HR: 0.49 (0.31–0.80), p = 0.003), sarcoma (SARC; HR: 0.49 (0.26–0.94), p = 0.032), and bladder urothelial carcinoma (BLCA; HR: 0.57 (0.37–0.86), p = 0.008) (Fig. 2C). These results are compatible with the role of glycolysis and OXPHOS in tumor progression [1], where tumors with higher rates of glycolysis but lower OXPHOS may be more aggressive than those with adverse features. Unexpectedly, LGHO was significantly associated with worse survival in esophageal carcinoma (ESCA; HR: 3.69 (1.32–10.31), p = 0.012), suggesting the potential distinct metabolic feature of ESCA. However, we failed to identify common prognostic value in HGHO and LGLO subtypes across multiple cancer types (Additional file 4: Fig. S4), thus, subsequent analyses were mainly focused on HGLO and LGHO.

Moreover, to further validate the metabolic discrepancy between HGLO and LGHO subgroups at a post-transcriptional level, we performed gene set enrichment analysis (GSEA) in 102 BRCA patients and ten OV patients, since only in these two tumor types the mass proteomic spectrum data were available in the CTPAC [9, 10]. The expression data of 9733 and 7625 proteins was available for BRCA and OV patients, respectively. Based on the metabolic group determined by RNA-sequencing data, the Glycolysis and OXPHOS pathway mentioned above also exhibited similar enrichment features for both cancer types (Glycolysis: adjusted p-value = 0.014 for BRCA and 0.004 for OV; OXPHOS: adjusted p-value = 0.121 for BRCA and 0.004 for OV, Fig. 2D). This phenomenon could be explained by the high correlations between mRNA and protein expression levels in a patient, further indicating the robustness of our metabolic scores and classification from another dimension.

### Association of metabolic subtypes with multi-omics genomic profiles across multiple cancer types

**Fig. 3:**
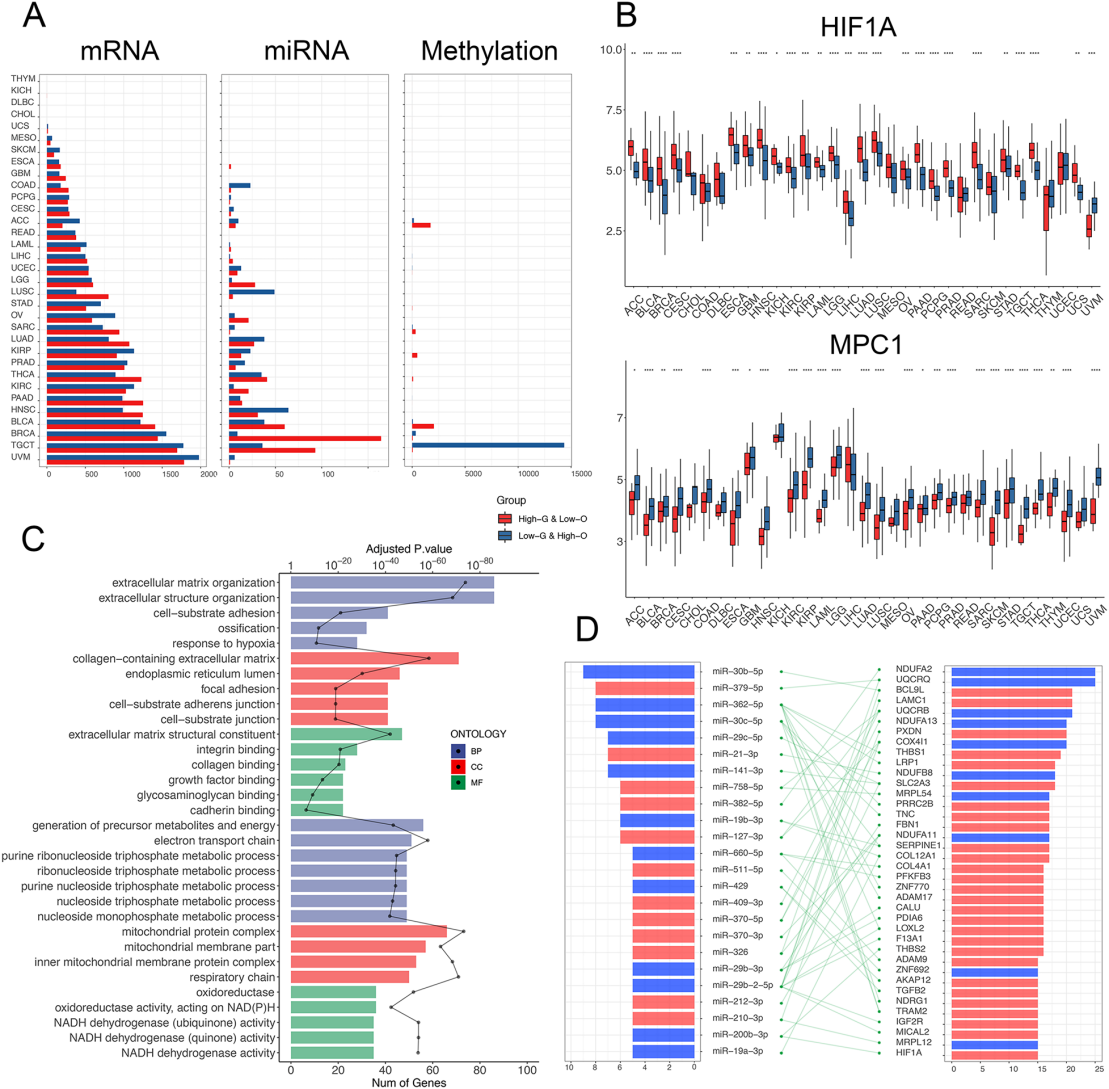
**A** Relative abundance and numbers of multidimensional significant discrepant metabolism-associated molecular features: mRNA, miRNA, and methylation differentially regulated between HGLO and LGHO across 33 cancer types. **B** Box plots showing the expression levels of HIF1A (upper) and MPC1 (lower) in HGLO and LGHO subgroup in different cancer types, respectively. Within each group, the scattered dots represent the two scores of each patient. The lines in the boxes represent the median value. The bottom and top of the boxes are the 25th and 75th percentiles (interquartile range). The whiskers encompass 1.5 times the interquartile range. The statistical difference of two scores was compared through the Wilcoxon test. *p < 0.05; **p < 0.01; ***p < 0.001; ****p < 0.0001. **C** GO functional enrichment analysis of the DEGs (upper 15 pathways for genes up-regulated in HGLO, lower 15 for LGHO). The bar plot indicates number of genes in each pathway, while the dotted lines displays the adjusted P-value of enrichment. BP, biological pathway; CC, cellular component; MF, molecular function. **D** Common differentially expressed miRNAs (left) and their potential target genes (right) across cancer types. The network (middle) shows the predicted targeting relationship between miRNAs and genes. The bilateral bar plots display the counts of cancer types in which miRNAs or genes are dysregulated (red: up-regulated in HGLO; blue: up-regulated in LGHO)

To systematically outline the genomic characteristics of patients in different metabolic subtypes and identify common changes or regulatory relationship across multiple cancer types, we performed a multi-omics comparison of three types of molecular features between the HGLO and LGHO groups in all 33 cancers: mRNA-sequencing, mature miRNA, and DNA methylation (Additional file 11: Data 3). Significant differential genomic features were detected and exhibited varied distribution across the 33 cancer types, which could be due to the distinct tumor characteristics and varied sample sizes (Fig. 3A). For instance, no DEG was detected in DLBC, cholangiocarcinoma (CHOL), and thymoma (THYM), while 3762 were detected in uveal melanoma (UVM). Differentially expressed miRNAs were identified in only 23 out of 33 cancers, and alterations in DNA methylation probes ranged from one in CESC to 14,405 in testicular germ cell tumors (TGCT).

Despite the considerable heterogeneity among different tumor types, we still noted lots of common significant genomic alterations across different cancer types, indicating the respective general characteristics of HGLO and LGHO subtypes, regardless of tumor origins. For example, our analysis revealed increased expression of NDUFA2 and UQCRQ, all key components of the mitochondrial respiratory chain, in LGHO group in 25/33 cancer types (Additional file 5: Fig. S5). The expression of MPC1, which encodes one subunit of mitochondrial pyruvate complex (MPC) that is responsible for transporting pyruvate into mitochondria, was downregulated in HGLO group in 15 cancer types (Fig. 3B). It has been reported that the effects of glycolysis on tumor progression can be diminished by diverting the metabolite pyruvate from conversion to lactate in part through transport into the mitochondria via the activity of the MPC1/MPC2 heterodimer [32–34]. Therefore, reduced expression of MPC1 is associated with increased glycolytic activity in tumors and thus promoting tumor development, which is consistent with our findings above. Besides, 15 tumor types exhibited increased expression of HIF1A, which is activated in hypoxia environment in tumor cells, in HGLO groups, indicating that hypoxia serves as the inducer of the glycolytic pathway and Warburg effect in cancer (Fig. 3B). Furthermore, GO enrichment analysis of the 632 genes (315 upregulated in HGLO and 317 upregulated in LGHO), which were differentially expressed in at least ten cancer types, also demonstrated similar results. As shown in Fig. 3C and Additional file 11: Data 3, in addition to the common pathways like extracellular matrix organization, we found the significant enrichment of a series of metabolic pathways involved in glycolysis and OXPHOS, including response to hypoxia (adjusted p-value < 0.001), respiratory chain (adjusted p-value < 0.001), and NADH dehydrogenase activity (adjusted p-value < 0.001), depicting the typical common genomic feature of different metabolic subgroups.

Considering the regulatory role of miRNA in gene expression, we also investigated the intersection between metabolism-related DEGs and differentially expressed miRNAs across multiple cancer types based on the miRNA-gene targeting relationship predicted by miRWalk (Additional file 12: Data 4). As shown in Fig. 3D, 24 key miRNAs (12 upregulated in HGLO and 12 in LGHO) and 38 genes (28 upregulated in HGLO and 10 in LGHO) were identified upon the following criteria: (1) the miRNAs are significantly differentially expressed in at least five tumor types; (2) their targets genes exhibit opposite expression mode in at least 15 tumor types. MiR-30c-5p (LGHO), miR-362-5p (LGHO), and miR-379-5p (HGLO) appear to be strong regulators of tumor glycolysis and OXPHOS since they were all dysregulated in 8 cancer types and targeted several metabolism-related DEGs. Meanwhile, the three miRNAs have been reported to play an important role in tumor metabolism and progression [35–37], which further supports our findings.

For differentially methylated genes (DMGs) between HGLO and LGHO subgroups, we focused on gene promoter region including TSS200, TSS1500, 3′-UTR, and 1st-Exon, since DMGs are commonly defined according to their promoter methylation status [38, 39]. The genes were classified into four groups based on the intersection between metabolism-associated DMGs and DEGs: hypermethylated and upregulated (hyper-up), hypermethylated, and downregulated (hyper-down), hypomethylated and upregulated (hypo-up), and hypomethylated and downregulated (hypo-down). Considering the nature of DMGs and DEGs, we focused on genes in the hyper-down and hypo-up groups in downstream analyses. However, these differentially methylated and expressed genes exhibited few generalities across tumor types, suggesting that abnormal methylation of a specific gene might not explain the metabolic alteration in multiple cancer types.

### Regulatory network of transcription factors in cancer metabolic heterogeneity

**Fig. 4:**
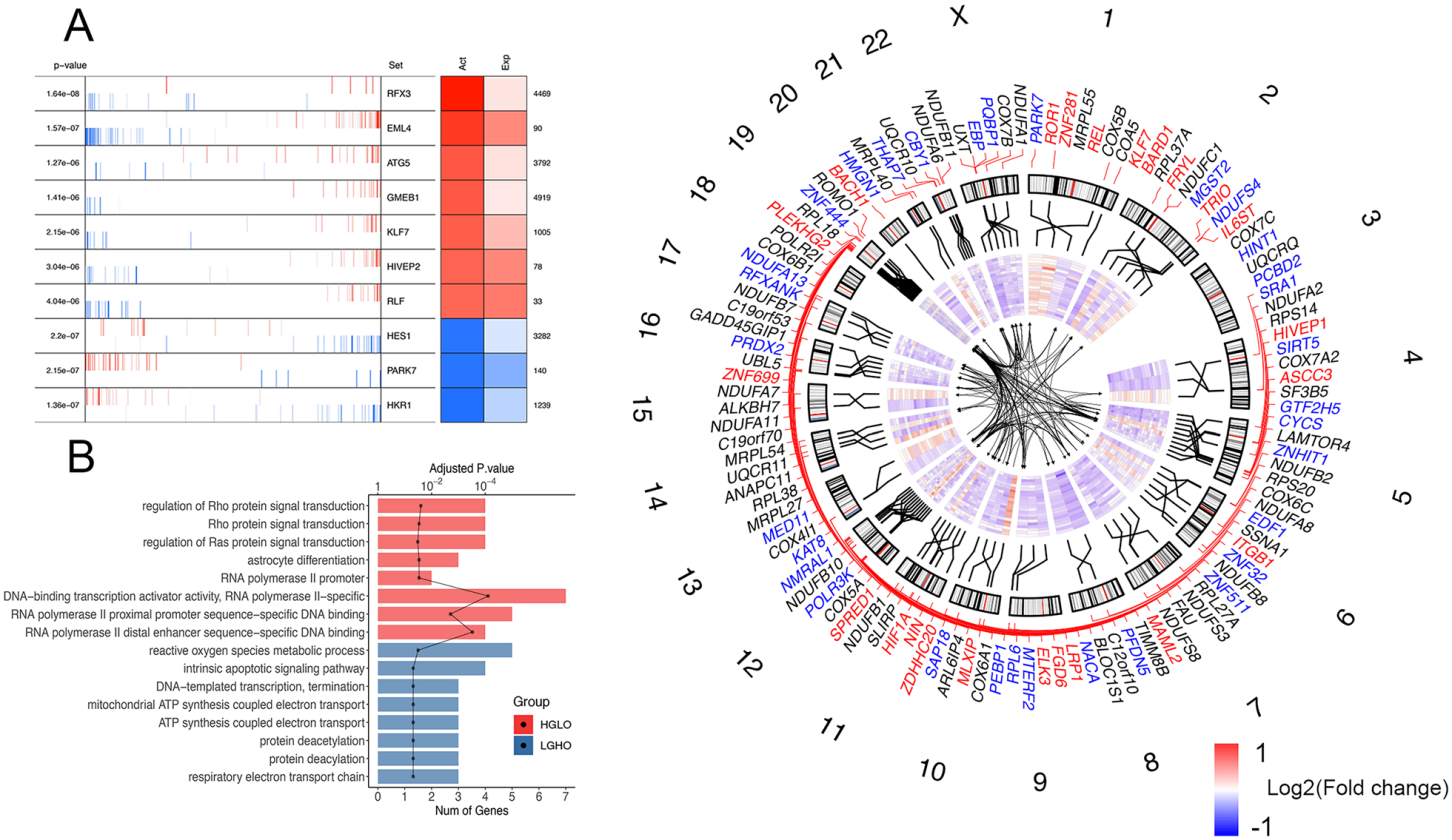
**A** The MARINa plot shows the projection of the negative (repressed, shown in blue color) and positive (activated, shown in red color) targets for each TF (vertical lines resembling a barcode) on the DEGs (x-axis) between HGLO and LGHO subgroups, where the DEGs were rank-sorted from the one most down-regulated to the on most upregulated in the HGLO vs LGHO conditions. Here we took BRCA as an example. The two-columns heatmap displayed on the right side shows the inferred differential activity (first column) and differential expression (second column) of each TF, with the rank of displayed genes in DEGs (shown on the right). The corresponding enrichment P-value are displayed on the left. **B** The GO functional enrichment analysis of the common TFs (upper 6 pathways for TFs activated in HGLO pathways, lower 6 for LGHO). The bar plot indicates number of genes in each pathway, while the dotted lines displays the adjusted P-value of enrichment. **C** Circos plot showing the 55 common TF and 56 common targets across cancer types. Outer to inner: (1) The number of chromosomes. (2) Red genes are the common TFs activated in HGLO group, while blue for LGHO and black for common targets. (3) Genomic location of TFs and targets. (4) Heatmaps showing the differential expression of TFs and targets across cancer types. Cancer types are GBM, LAML, THYM, THCA, TGCT, READ, PRAD, PCPG, PAAD, OV, LUSC, LUAD, LIHC, KIRP, KIRC, HNSC, ESCA, COAD, CESC, BRCA, and BLCA (ordered outer to inner). (5) Networks displaying the regulatory network of these common TFs and targets

To further elucidate how the tumor metabolic status is regulated by transcription factors (TFs), we adopted tumor-context-specific TF regulatory networks, which is based on ARACNe algorithm and proposed by Giorgi et al. [18], to infer the significantly enriched master TFs that potentially drive the conversion of tumor metabolic phenotypes (HGLO and LGHO) from one to the other using MARINa in each cancer type (Additional file 13: Data 5). The results were further statistically adjusted by shadow analysis [19] and the activating and expression level of identified TFs in different cancer types are shown in Fig. 4A (we took BRCA as an example) and Additional file 6: Fig. S6. After this two-step screening method, 55 common TFs (see “Methods”) were enrolled for subsequent analyses, and the result of functional enrichment of them also confirms their role in multiple metabolic pathways, including respiratory electron transport chain (Fig. 4B). We then employed leading-edge analysis to identify the 56 target genes of those common TFs (Additional file 13: Data 5) and integrated their genomic location, relative expression level across different cancer types, and corresponding TF-target regulatory network in Fig. 4C. For example, MTERF2, which has been demonstrated to be involved in mitochondrial gene transcription and metabolism and serve pivotal roles in the pathogenesis of various cancer types [40], was identified as a master TF driving the LGHO phenotype in 7 cancer types. The regulatory network analysis revealed that ZNF10, a transcriptional repressor protein, was MTERF2’s potential target in 10 cancers. Meanwhile, we found that HIF1A, which is up-regulated in 15 tumor types as mentioned above, served as a master TF for the HGLO phenotype in 7 of them, as tumor formation associated gene SNAPC1 was identified as its downstream target in 6 cancers [41]. In addition, we noticed an interesting phenomenon that many TFs mentioned above are also downstream target genes of other TFs, indicating the complicated TF-related regulatory network of tumor metabolism status.

### Metabolism associated somatic genomic changes

**Fig. 5:**
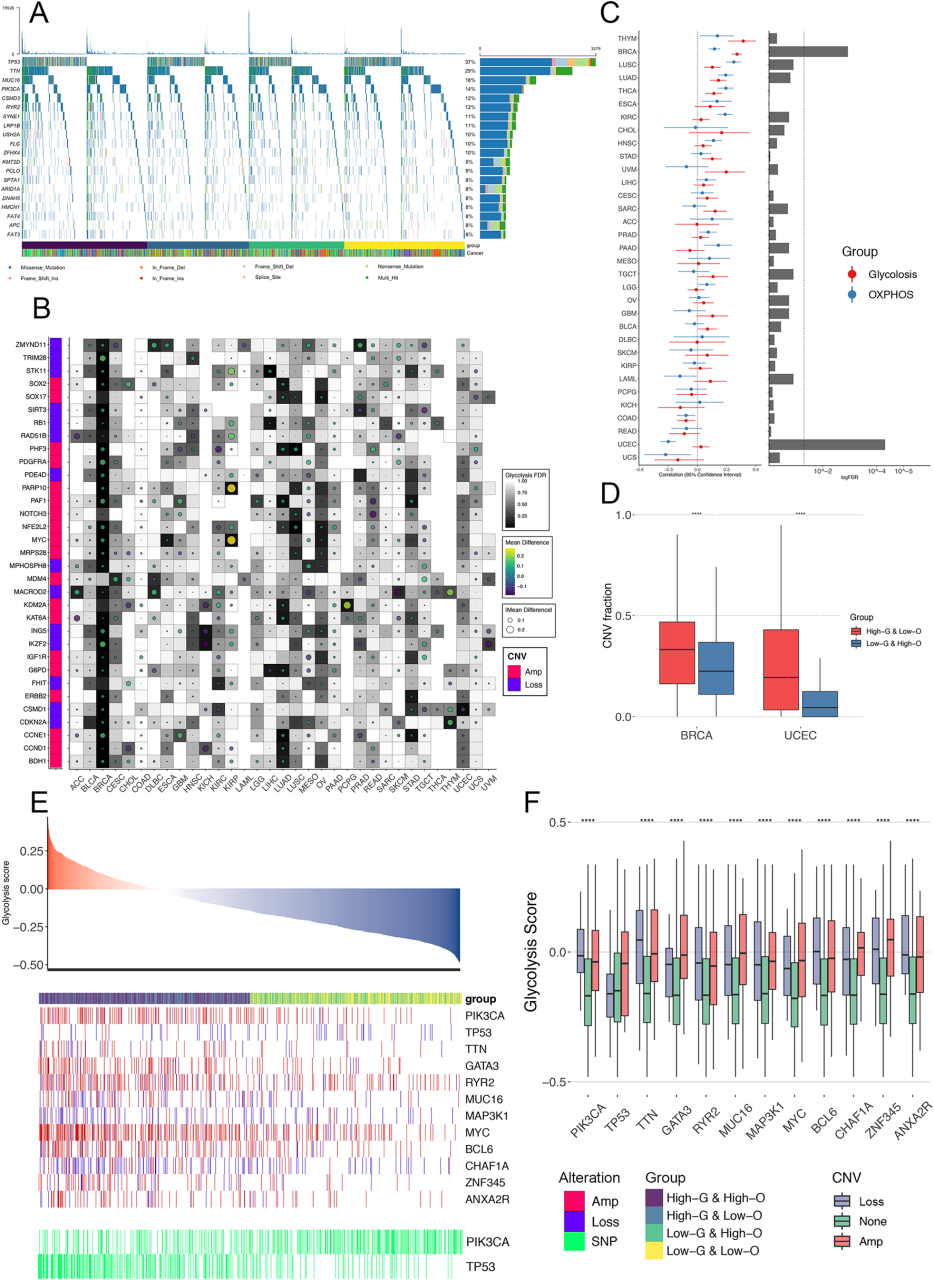
**A** The waterfall plots summarizing the somatic SNVs of 20 most-frequently mutated genes in the four metabolic subgroups across 33 cancer types. The type of alterations was annotated by different colors (bottom). **B** Association of Glycolysis score with CNVs in oncogenes (labeled as red on the left) and tumor suppressor genes (labeled as blue on the left, Wilcoxon test). Dot size and color indicate the difference in mean Glycolysis between tumors with a CNV (gain for oncogene and loss for tumor suppressor gene) and those without. Background color indicates the FDR of Wilcoxon test. The empty region indicates that in that cancer type, there is no CNV of a specific gene. **C** Left: The correlation between CNV fraction and two pathway scores. Center dots represent Pearson’s r, and error bars represent the 95% CI. Right: FDR were calculated by Wilcoxon test to compare the CNV fraction between HGLO and LGHO group across multiple cancer types. **D** Box plots displaying the distribution of CNV fraction between HGLO and LGHO group in BRCA and UCEC. Within each group, the lines in the boxes represent the median value. The bottom and top of the boxes are the 25th and 75th percentiles (interquartile range). The whiskers encompass 1.5 times the interquartile range. The statistical difference of two scores was compared through the Wilcoxon test. ****p < 0.0001. **E** Associations of SNVs and CNAs with Glycolysis score in BRCA patients. **F** Box plots displaying the distribution of Glycolysis score among patients with different CNV status (amplification, loss, and none) in BRCA. Within each group, the lines in the boxes represent the median value. The bottom and top of the boxes are the 25th and 75th percentiles (interquartile range). The whiskers encompass 1.5 times the interquartile range. The statistical difference of two scores was compared through the Wilcoxon test. ****p < 0.0001

The reprogramming of the glycolytic and OHPHOS status can be largely viewed as a consequence of oncogenic driver events [42]. For instance, it has been widely accepted that mutated TP53 and amplified MYC are linked to several anabolic and catabolic pathways [43, 44]. To identify somatic genomic changes that potentially characterize tumor metabolism, we systematically evaluated the association between metabolic subtypes and specific mutational events, including both SNVs and CNVs. For SNVs, Fig. 5A shows the overall mutational landscape of the 15 most frequently mutated genes on the pan-cancer scale (Additional file 14: Data 6). Besides, for each cancer type, we identified significantly differently mutated genes and only noted the elevation rated of TP53 (Fisher-exact test, P < 0.05) across five cancer types in HGLO compared with LGHO, which is in agreement with its reported function in metabolism and the selection of TP53-mutated cells under hypoxic stress [45]. However, most metabolism-related SNVs were detected in only one or two tumor types, thus highlighting strong inter-tumor-type diversity in this mutational feature.

As for CNVs, we first focused on 131 cancer driver genes altered by CNVs [22] and identified the oncogenes and tumor suppressors recurrently associated with tumor metabolism in multiple cancer types (Fig. 5B; Additional file 7: Fig. S7A). For example, the amplification of the oncogene MYC was significantly associated with elevated Glycolysis score in four independent cancer types (FDR < 0.05). However, we failed to find CNVs recurrently associated with OXPHOS in even three cancer types, indicating that individual tumor types might have distinctive cancer driver CNV-metabolism relationships. We next investigated the CNV fraction, a surrogate of genomic instability that is correlated with aggressivity in several tumor types (Fig. 5C) [46, 47]. Our analysis revealed that CNV fraction was significantly different between HGLO and LGHO subtypes only in BRCA and uterine corpus endometrial carcinoma (UCEC, FDR adjusted Wilcoxon P < 0.05, Fig. 5C, D). Meanwhile, strong correlations were observed between CNV fraction value and the Glycolysis score in BRCA and the OXPHOS score in UCEC, respectively. (Fig. 5C, left). Therefore, we focused on these two cancers and further assessed whether metabolic scores were associated simply with specific gene-level mutational events, including both CNVs and SNVs. As shown in Fig. 5E, F, in BRCA, tumors harboring amplification of PIK3CA, GATA3, MYC, and deletion of MAP3K1, CHAF1A are more likely to have higher Glycolysis score. Besides, high glycolytic breast tumors also exhibit an elevated rate of TP53 (OR = 3.18, P < 0.001) and decreased rate of PIK3CA point mutation (OR = 0.36, P < 0.001). As for UCEC, the strong association between mutational events and OXPHOS score was observed. Tumors with amplification of PIK3CA and BCL6, or those with deletion of ARID1A and CH1F1A, are more likely to show lower OXPHOS score (Additional file 7: Fig. S7B, C). Additionally, TP53 point mutations are more common in low-OXPHOS tumors (OR = 2.63, P < 0.001). Taken together, these analyses provide a broad view of potential somatic drivers related to metabolic reprogramming in human cancer, thereby proposing testable hypotheses for future experimental modeling studies.

### Tumor metabolism are informative about pathway functional enrichment and drug response

**Fig. 6:**
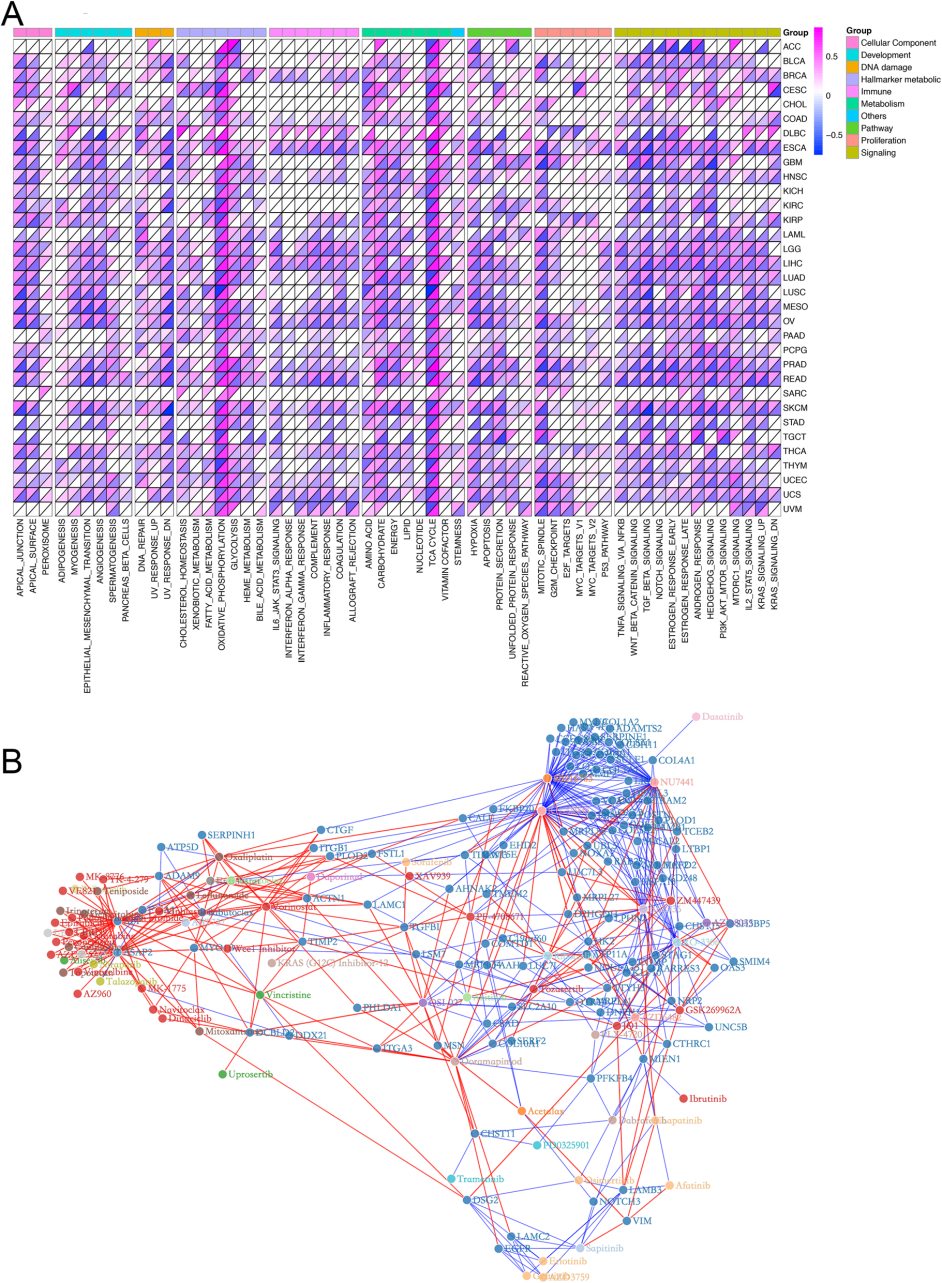
**A** Glycolysis (upper part of each grid) and OXPHOS (lower part of each grid) scores’ correlations with other pathways across cancer types. **B** The network exhibits the correlation between the drug sensitivity in cancer cell lines from GDSC and the expression level of 438 metabolism-associated DEGs across cancer types. Dark blue dots represent genes, while dots with other colors represent the anti-cancer drugs targeting different biological pathways. Red lines indicate positive correlation and blue lines indicate negative

To further assess the biological relevance of metabolic subtypes, we examined their correlation with various cellular pathways based on mRNA expression (FDR < 0.05, Fig. 6A). This analysis enrolled the 50 hallmark gene sets proposed by Liberzon et al. [23] and seven metabolic super-pathways from the latest Reactome annotations [24]. The GSVA score of these 57 pathways was calculated for each cancer patient. Tumor stemness data proposed by Malta et al. was also enrolled [25]. Interestingly, despite the diversity of cancer types surveyed, we observed an almost adverse correlation pattern of most pathway enrichment levels with Glycolysis and OXPHOS score across multiple cancer types. For example, the majority of included pathways, including angiogenesis, epithelial-mesenchymal transition, TGF-beta signaling, hypoxia, and mTORc, were consistently positively correlated with Glycolysis but negatively correlated with OXPHOS score. In contrast, asides from the TCA cycle, which is an important section of OXPHOS process, the tumor-stemness and amino acid metabolism pathway displayed recurrent positive correlations with OXPHOS, but negative correlation with Glycolysis score across several tumor types. Overall, these results demonstrate that tumor metabolic activity is intrinsically coupled with cancer pathways and development.

Considering the previously reported associations of drug resistance with tumor metabolic features [30], we adopted the expression and drug sensitivity data of cancer cell lines from GDSC and investigated whether the tumor cells from HGLO & LGHO subtypes would harbor different sensitivity to anti-cancer drugs. The RNA sequencing data of 435 cancer cell lines and their sensitivity to 167 anti-cancer drugs, presented as AUC, were used for subsequent analyses. As shown in Additional file 8: Fig. S8, some of the commonly used anti-cancer drugs, including cisplatin, gemcitabine, and paclitaxel, as well as mTOR suppressor OSI_027, exhibited significant different pharmacological effect in the two metabolic subtypes. Besides, we further analyzed the correlation between drug sensitivity and the expression of 438 metabolism-associated DEGs, which were significantly differentially expressed between HGLO and LGHO subgroups in at least ten cancer types. These drugs target several important biological processes, including metabolism, apoptosis, DNA replication, PI3K-mTOR signaling, and the p53 pathway. The network in Fig. 6B depicts the complicated correlations among the anti-cancer drugs and 111 of 438 metabolism-related DEGs that were strongly correlated (|r|> 0.3) with cell line sensitivity (AUC) to at least three drugs. For example, the expression of ASAP2, which has been identified as a biomarker for several types of tumor, is up-regulated in HGLO in 16 cancer types and positively correlated with the resistance to DNA topoisomerases inhibitor Topotecan (r = 0.40) and DNA synthesis inhibitor Gemcitabine (r = 0.34). It has been reported that overexpression of ASAP2 is correlated with worse prognosis in pancreatic cancers [48]. Besides, Overexpression of APP in HGLO group was detected in 15 cancer types and suggested potential resistance to 33 types of drugs, including Teniposide, Mitoxantrone, Oxaliplatin, and Irinotecan, all of the which target DNA replication (r = 0.434, r = 0.425, r = 0.416, and r = 0.366, respectively). Taken together, our findings demonstrate the extensive interactions between drug responses and tumor metabolism, suggesting that our metabolic classification might serve as a predictive marker for the response to different anti-cancer treatment.

## Discussion

Metabolic reprogramming is considered a hallmark of cancer and has been an area of accelerated research over the last decade [23, 49, 50]. Although metabolic gene expressional levels are not perfectly equivalent to metabolic fluxes or metabolite abundance, our analysis on a BRCA cohort with parallel metabolite and gene expression data demonstrated that metabolic gene profiling data could to some if not a large extent reflect metabolic activities, which has also been certified by previous researchers [30, 51]. Based on the profiling data of genes involved in glycolysis and OXPHOS, we developed a “double-score” system and classified the 9668 patients spanning 33 tumor types into four metabolic subtypes, thus depicting the clinical and genomic features of tumors with distinct functional status from multiple dimensions. Through our analyses, several potential drivers or master regulators associated with tumor metabolic rewiring were identified, which may guide therapeutic strategies for cancer patients in the future.

There is still a controversy on the mutual relationship between glycolysis and OXPHOS in tumor. Our analysis on the scores of these two pathways across different cancer types exhibited extensive intertumor- and intratumor-type heterogeneous distribution pattern: the activity of these two pathways is not completely opposed to the other as expected. However, we even noticed some kinds of positive correlation in both pan-cancer scale and several independent cancer types (THYM: r = 0.573, FDR < 0.001; TCGT: r = 0.564, FDR < 0.001). Meanwhile, in addition to the long-standing notion that tumors are primarily glycolytic, including under aerobic conditions, as postulated by the Warburg effect, several recent studies have demonstrated OXPHOS system upregulation in many tumors and is a potential therapeutic target [52, 53]. Several oncogenic pathways rely on mitochondrial metabolism and OXPHOS plays a critical role in tumor progression and aggressiveness at every stage of development [54]. For instance, in cholangiocarcinoma, OXPHOS system contributes to a cancer stem cell phenotype and correlates with patients’ clinical outcome [55]. Besides, LAML cells have an atypical metabolic phenotype characterized by great reliance on OXPHOS [56, 57]. These findings suggested that the alteration in the balance between glycolysis and OXPHOS is more than simple “switch-on/off,” leading to a growing interest in translating this information into clinical practice for prognostication and treatment response prediction. Survival analysis on the four metabolic subtypes indicated that HGLO subtype is a negative prognostic factor in multiple cancer types, compared with LGHO, while HGHO and LGLO failed to exhibit typical prognostic value, which is in agreement with previously reported role of glycolysis and OXPHOS in tumorigenesis and progression [4, 42, 50, 58, 59]. Therefore, we inferred that tumor patients with HGLO or LGHO subtypes are more likely to have worse or better survival respectively, and the drivers or targets motivating this transition might harbor potential therapeutic value, thus benefiting selected tumor patients.

In our study, we identified a series of molecular alterations showing consistency as pan-cancer correlates of metabolic status, as well as several differentially enriched metabolism-associated biological pathways. In addition to MPC1, the “intersection point” between glycolysis and OXPHOS, we also found the increased expression of lactate dehydrogenase A (LDHA), an enzyme catalyzing the conversion of pyruvate and lactate thus promoting glycolysis and suppressing OXPHOS, in HGLO subgroup across cancer types [60]. Meanwhile, many of its up-stream molecules, such as HIF1a and mTOR, also exhibited consistent alteration tendency, which is in agreement with previous researches. It has been reported that HIF1a, which could be activated by hypoxia or oncogenic signaling (for example, downstream of mTORC1) pathways [61, 62], is an important contributor to the Warburg effect through various mechanisms: (1) inducing the expression of genes encoding glycolytic enzymes and glucose transporters [63–65]; (2) suppressing OXPHOS by inducing the expression of pyruvate dehydrogenase kinase 1 (PDK1), which phosphorylates and inhibits mitochondrial pyruvate dehydrogenase (PDH) [63, 66, 67]. These findings were further supported by our findings that Glycolysis score was positively correlated with that of both hypoxia and mTORc signaling pathway, while OXPHOS score exhibited the opposite result. As for others, such as somatic alterations, our result that the amplification of oncogene MYC and mutation of tumor suppressor gene TP53 are enriched in HGLO supports the idea that they were regarded as the driver of the initiation of tumor metabolic reprogramming [43, 44, 60]. Meanwhile, as shown in the results from functional enrichment analyses, several metabolic pathways associated with hypoxia, glycolysis, and OXPHOS were enriched based on the differentially expressed genes between HGLO and LGHO subgroups. Therefore, it is natural to speculate that the metabolic reprogramming is tightly intersected with the dysregulation of these genes.

A series of potential therapeutic targets were identified through our comprehensive analysis of common DEGs in multiple cancer types and drug-response data, such as the “core nodes” in Fig. 6B, like ASAP2, APP, and ACTN1, whose expression level were found to be significantly correlated to cell’s response to several different anti-cancer drugs targeting different pathways. For example, one of the “core” drugs, the ATM kinase inhibitor KU55933, has been demonstrated to induces apoptosis and inhibits motility by blocking GLUT1-mediated glucose uptake in aggressive cancer cells [68]. Besides, we also observed vast metabolism-associated dysfunction of miRNAs and TF across cancers. Further efforts will be warranted to experimentally determine the clinical relevance of these genomic features in patients with different metabolic characteristics.

However, our study still has several limitations. First, due to the lack of accessible metabolites data in published studies or open-access database, we failed to comprehensively analyze the correlation between metabolites and metabolic proteins, especially mitochondrial proteins, since only the data of 3 metabolites involved in OXPHOS were provided. And as for GSEA analyses based on proteomic data, only the data of BRCA and OV patients were available in TCGA database, limiting us from further validating our results by GSEA in other cancer types. Besides, it is difficult for us to disentangle the timing of these events: whether a specific gene alteration leads to metabolic change or results from metabolic reprogramming. Meanwhile, considering the spatial heterogeneity in one tumor sample, the lack of multi-loci sampling RNA-sequence data within a single tumor in these public large-scale data sets like TCGA might weaken the predictive value of our metabolic subtypes. With the rapid development of novel single-cell omics techniques, we are optimistic that a dynamic and comprehensive portrait of tumor metabolism will emerge in the near future. Further in vitro and in vivo experiments are warranted to validate our findings in another dimension.

## Conclusions

By integrating multi-omics data, our large-cohort pan-cancer study provides a comprehensive atlas of genomic factors associated with tumor metabolic reprogramming and extracts the common molecular alterations across tumor types, thus shedding light on the complex regulatory network of tumor’s metabolic status and may guide a more precise and personalized therapeutic strategy for tumor patients with metabolic reprogramming.

## Supplementary Information

**Additional file 1: Fig. S1. **Overview of the study design.

**Additional file 2: Fig. S2. (A)** Correlation between the Glycolysis and OXPHOS scores of all patients enrolled in this study. **(B-E)** The scatter plot exhibiting the correlation between the Glycolysis **(B, C, D)** or OXPHOS **(E)** score and abundance of metabolites.

**Additional file 3: Fig. S3.** Kaplan–Meier curves showing the prognostic value of four metabolic subgroups in 33 cancer types from TCGA.

**Additional file 4: Fig. S4.** Forest plots showing the pairwise survival analysis among four metabolic subgroups in each cancer type, with corresponding HR (log-transformed) and 95%CI (log-transformed).

**Additional file 5: Fig. S5.** Box plots showing the expression levels of NDUFA2 (upper) and UQCRQ (lower) in HGLO and LGHO subgroup in different cancer types respectively. Within each group, the scattered dots represent the two scores of each patient. The lines in the boxes represent the median value. The bottom and top of the boxes are the 25^th^ and 75^th^ percentiles (interquartile range). The whiskers encompass 1.5 times the interquartile range. The statistical difference of two scores was compared through the Wilcoxon test. *, p < 0.05; **, p < 0.01; ***, p < 0.001; ****, p < 0.0001.

**Additional file 6: Fig. S6.** Heatmap displaying the Normalized Enrichment Score (NES, shown as background color) and FDR (shown as dot size and color) of common TFs in different cancer types. Only TFs with an FDR < 0.05 are shown in the plot.

**Additional file 7: Fig. S7. (A)** Association of OXPHOS score with copy number variation (CNVs) in oncogenes (labeled as red on the left) and tumor suppressor genes (labeled as blue on the left, Wilcoxon test). Dot size and color indicate the difference in mean OXPHOS between tumors with a CNV (gain for oncogene and loss for tumor suppressor gene) and those without. Background color indicates the FDR of Wilcoxon test. The empty region indicates that in that cancer type, there is no CNV of a specific gene. **(B)** Associations of SNVs and CNAs with OXPHOS score in UCEC patients. **(C)** Box plots displaying the distribution of OXPHOS score among patients with different CNV status (amplification, loss, and none) in UCEC. Within each group, the lines in the boxes represent the median value. The bottom and top of the boxes are the 25^th^ and 75^th^ percentiles (interquartile range). The whiskers encompass 1.5 times the interquartile range. The statistical difference of two scores was compared through the Wilcoxon test. ****, p < 0.0001.

**Additional file 8: Fig. S8.** Box plots displaying the distribution of AUC value, which reflects the sensitivity to specific anti-cancer drug, among cancer cells in different metabolic group. Within each group, the lines in the boxes represent the median value. The bottom and top of the boxes are the 25^th^ and 75^th^ percentiles (interquartile range). The whiskers encompass 1.5 times the interquartile range. The statistical difference of two scores was compared through the Wilcoxon test. *, p < 0.05; **, p < 0.01; ***, p < 0.001.

**Additional file 9: Data 1.** Gene signatures mentioned in the study for GSVA analysis.

**Additional file 10: Data 2.** Invasiveness score of the 9668 patients from 33 cancer types in TCGA.

**Additional file 11: Data 3.** Metabolism-associated molecular features across 33 cancer types. (HGLO vs. LGHO).

**Additional file 12: Data 4.** The potential target genes of metabolism-related differentially expressed miRNA across 33 cancer types.

**Additional file 13: Data 5.** Metabolism-associated transcriptive factors and their potential targets across 33 cancer types.

**Additional file 14: Data 6.** Metabolism-associated somatic single nucleotide variants across 33 cancer types.

## Data Availability

All data generated or analyzed during this study are included in this published article and its supplementary information files. Source data were obtained from TCGA database.

## Abbreviations

OXPHOS: Oxidative phosphorylation
TCGA: The cancer genome atlas
CNVs: Copy number variation
BRCA: Breast invasive carcinoma
OV: Ovarian serous cystadenocarcinoma
CTPAC: Clinical proteomic tumor analysis consortium
ssGSEA: Single-cell gene set enrichment analysis
FDR: False discovery rates
DEGs: Differentially expressed genes
TF: Transcription factor
MARINa: Master regulator inference algorithm
GO: Gene ontology
DLBC: Lymphoid neoplasm diffuse large B-cell
LAML: Lymphoma acute myeloid leukemia
COAD: Colon adenocarcinoma
LIHC: Liver hepatocellular carcinoma
READ: Rectum adenocarcinoma
KICH: Kidney chromophobe
HGHO: High-glycolysis and high OXPHOS
HGLO: High-glycolysis and low OXPHOS
LGHO: Low-glycolysis and high OXPHOS
LGLO: Low-glycolysis and low OXPHOS
LUAD: Lung adenocarcinoma
CESC: Cervical squamous cell carcinoma and endocervical adenocarcinoma
KIRP: Kidney renal papillary cell carcinoma
MESO: Mesothelioma
PAAD: Pancreatic adenocarcinoma
SARC: Sarcoma
BLCA: Bladder urothelial carcinoma
ESCA: Esophageal carcinoma
CHOL: Cholangiocarcinoma
THYM: Thymoma
UVM: Uveal melanoma
TGCT: Testicular germ cell tumors
MPC: Mitochondrial pyruvate complex
DMGs: Differentially methylated genes
PDK1: Pyruvate dehydrogenase kinase 1
PDH: Pyruvate dehydrogenase
