# Supplementary figures and images for: Pan-cancer characterization of metabolism-related biomarkers identifies potential therapeutic targets
Source: J Transl Med. 2021 May 24;19:219. doi: 10.1186/s12967-021-02889-0 (PMC8142489; doi:10.1186/s12967-021-02889-0)

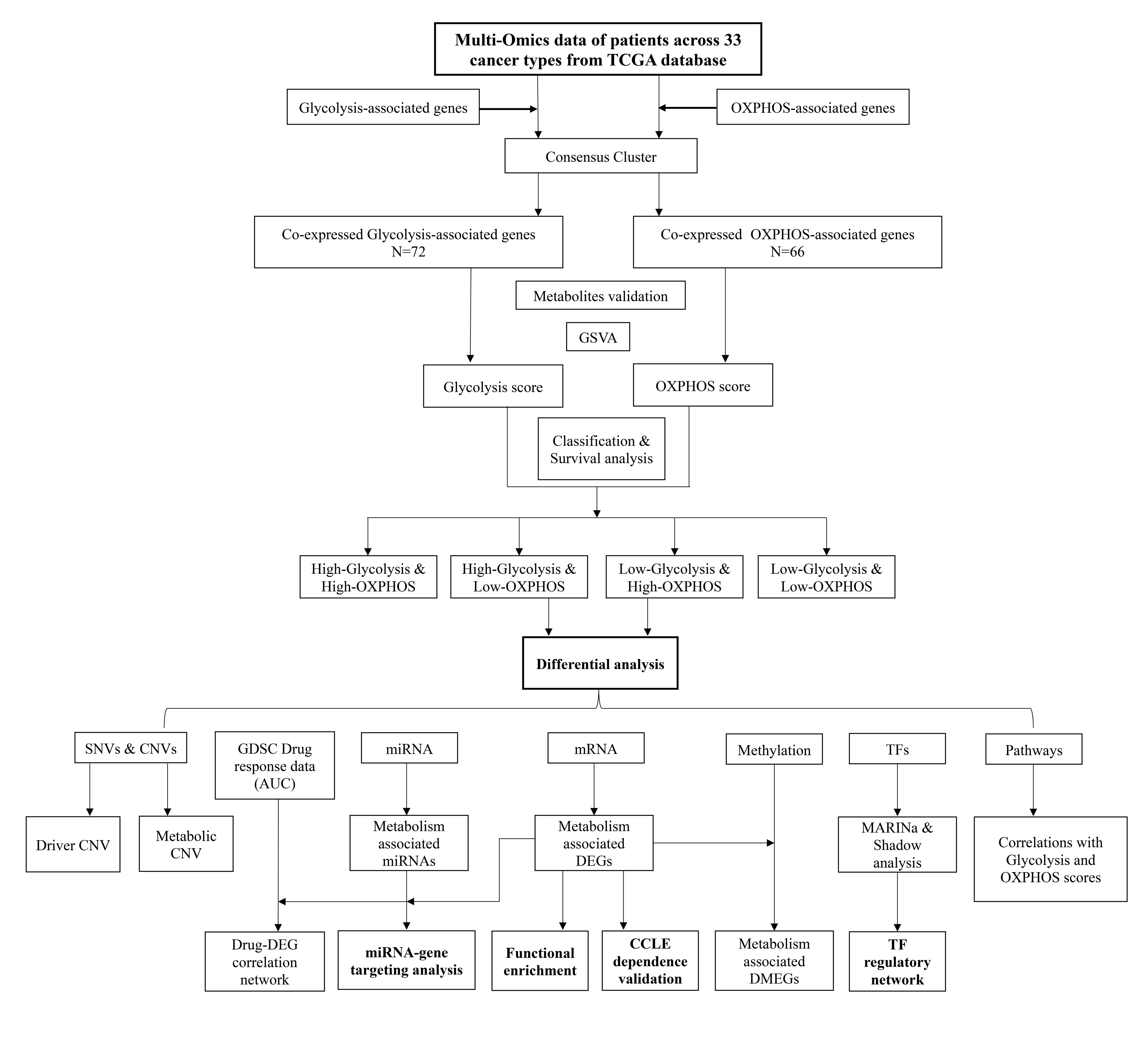

Supplement: Supplementary file 1 — Additional file 1: Fig. S1. Overview of the study design. [file 12967_2021_2889_MOESM1_ESM.png]

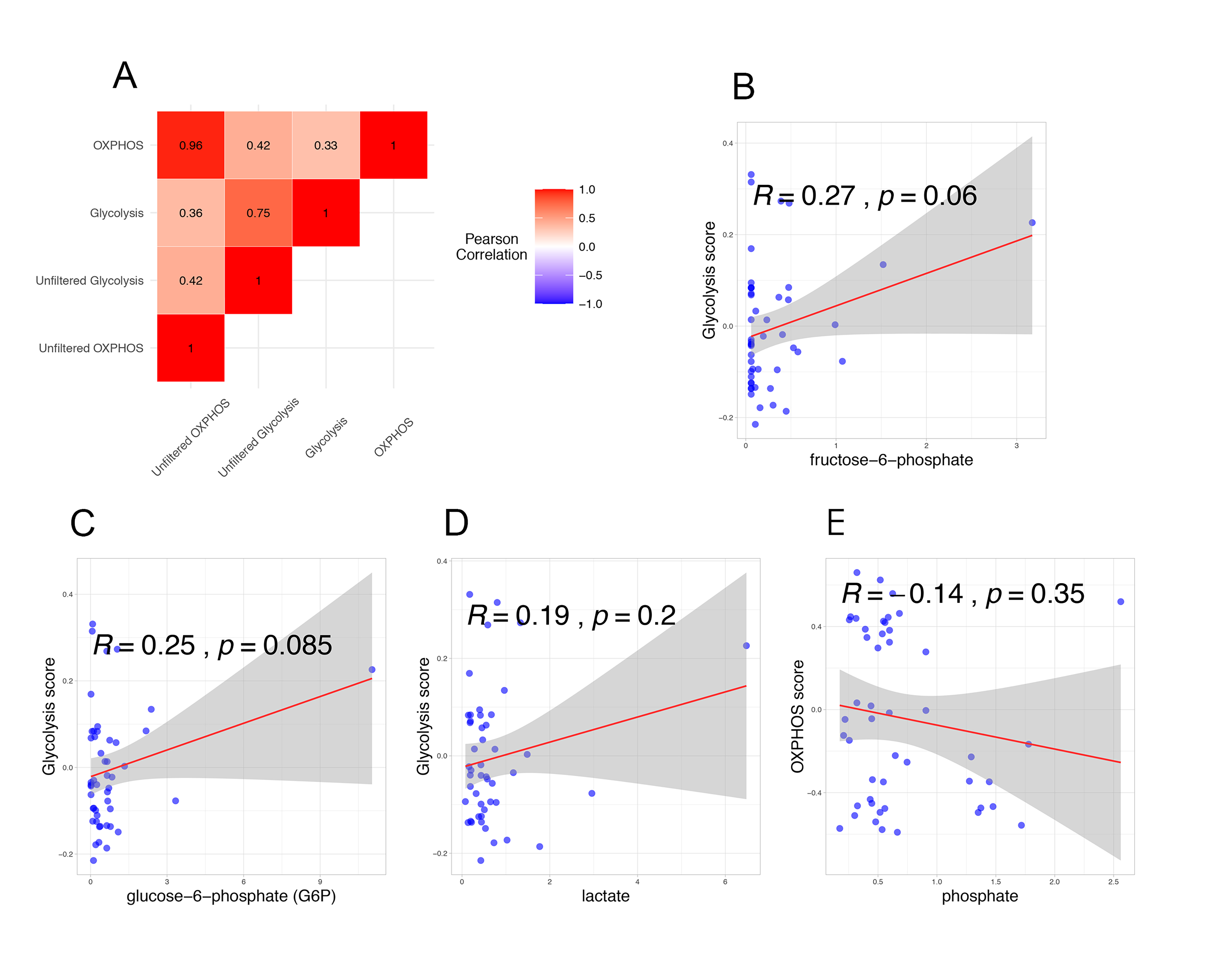

Supplement: Supplementary file 2 — Additional file 2: Fig. S2. (A) Correlation between the Glycolysis and OXPHOS scores of all patients enrolled in this study. (B-E) The scatter plot exhibiting the correlation between the Glycolysis (B, C, D) or OXPHOS (E) score and abundance of metabolites. [file 12967_2021_2889_MOESM2_ESM.png]

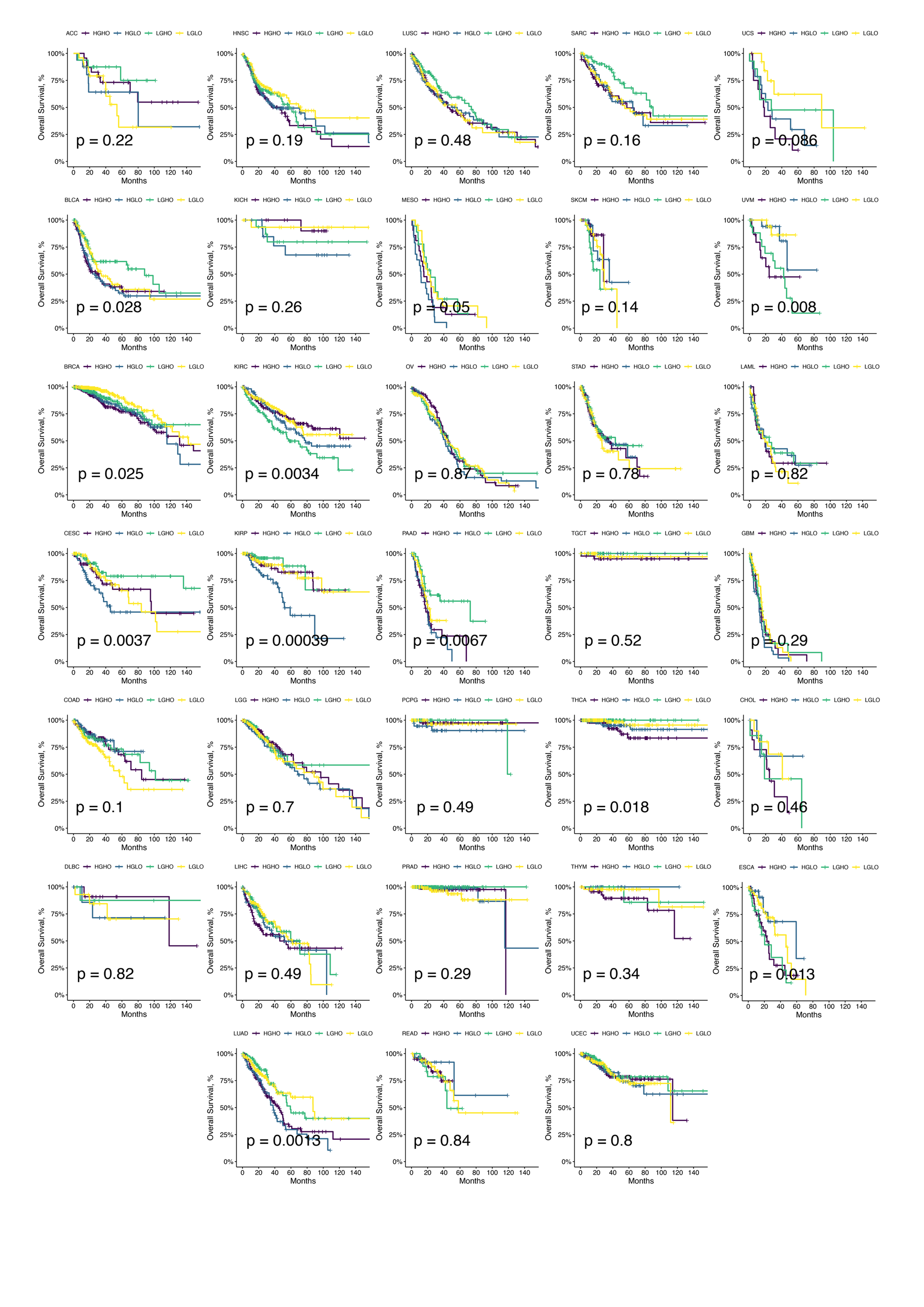

Supplement: Supplementary file 3 — Additional file 3: Fig. S3. Kaplan–Meier curves showing the prognostic value of four metabolic subgroups in 33 cancer types from TCGA. [file 12967_2021_2889_MOESM3_ESM.png]

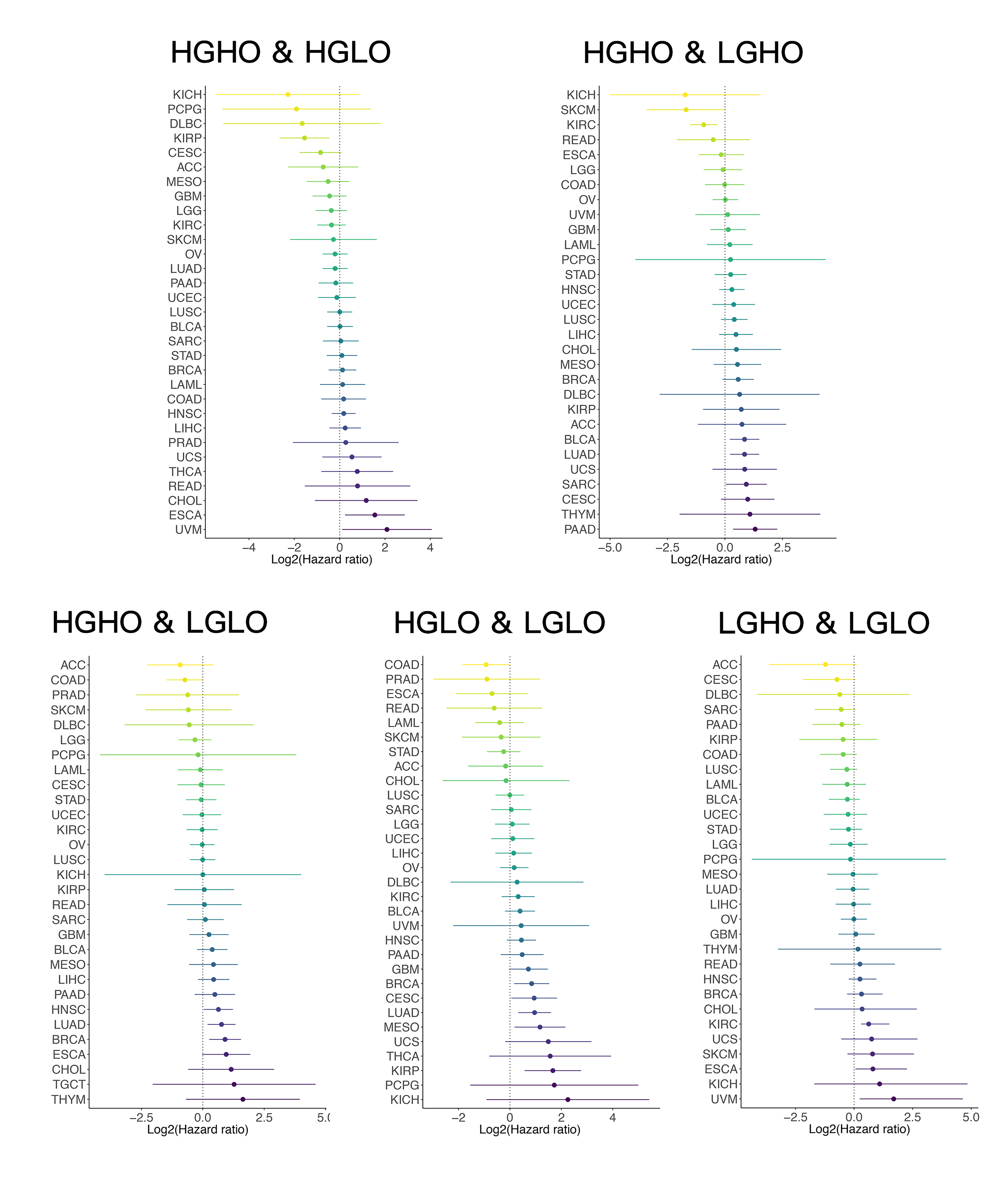

Supplement: Supplementary file 4 — Additional file 4: Fig. S4. Forest plots showing the pairwise survival analysis among four metabolic subgroups in each cancer type, with corresponding HR (log-transformed) and 95%CI (log-transformed). [file 12967_2021_2889_MOESM4_ESM.png]

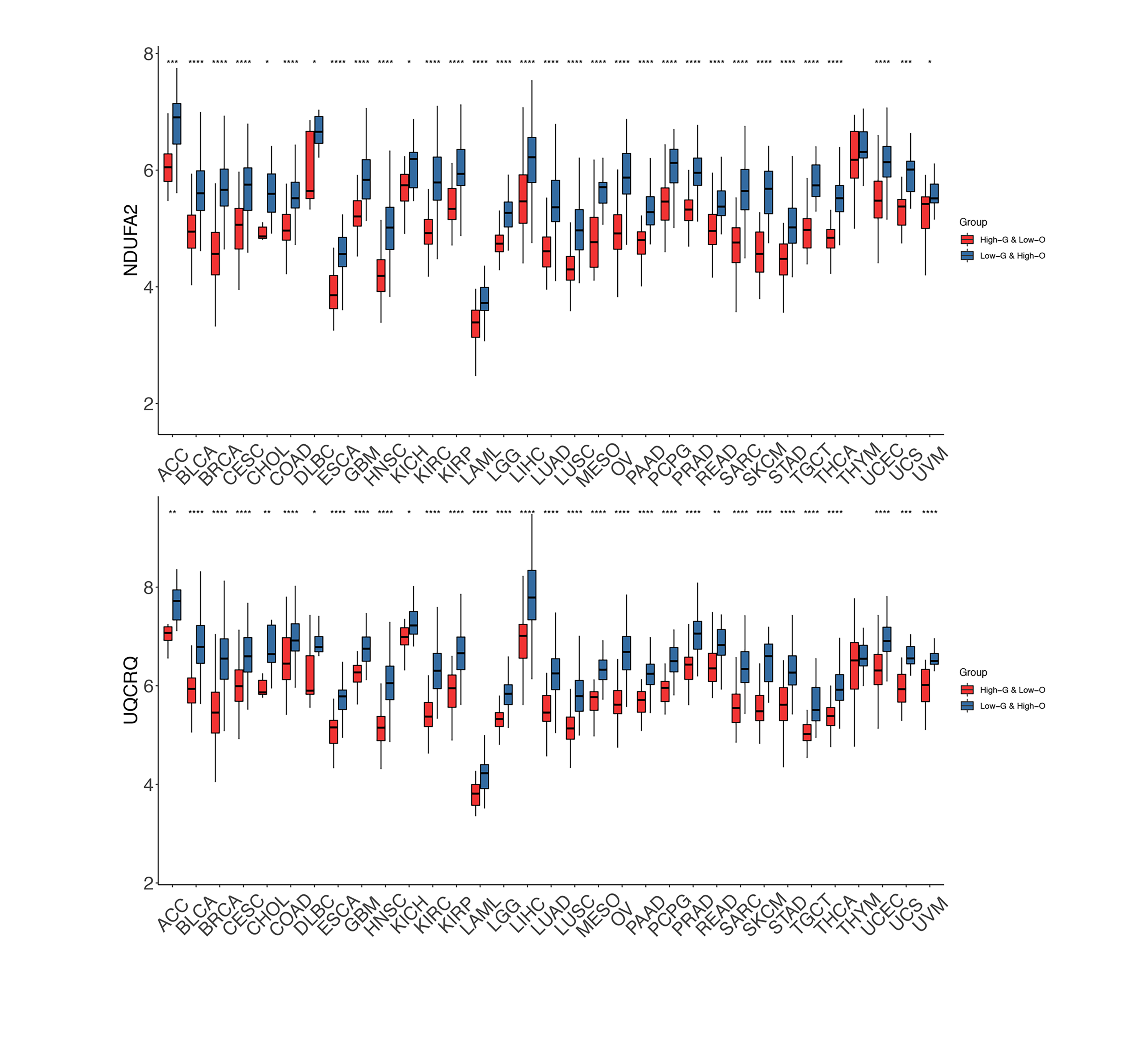

Supplement: Supplementary file 5 — Additional file 5: Fig. S5. Box plots showing the expression levels of NDUFA2 (upper) and UQCRQ (lower) in HGLO and LGHO subgroup in different cancer types respectively. Within each group, the scattered dots represent the two scores of each patient. The lines in the boxes represent the median value. The bottom and top of the boxes are the 25th and 75th percentiles (interquartile range). The whiskers encompass 1.5 times the interquartile range. The statistical difference of two scores was compared through the Wilcoxon test. *, p < 0.05; **, p < 0.01; ***, p < 0.001; ****, p < 0.0001. [file 12967_2021_2889_MOESM5_ESM.png]

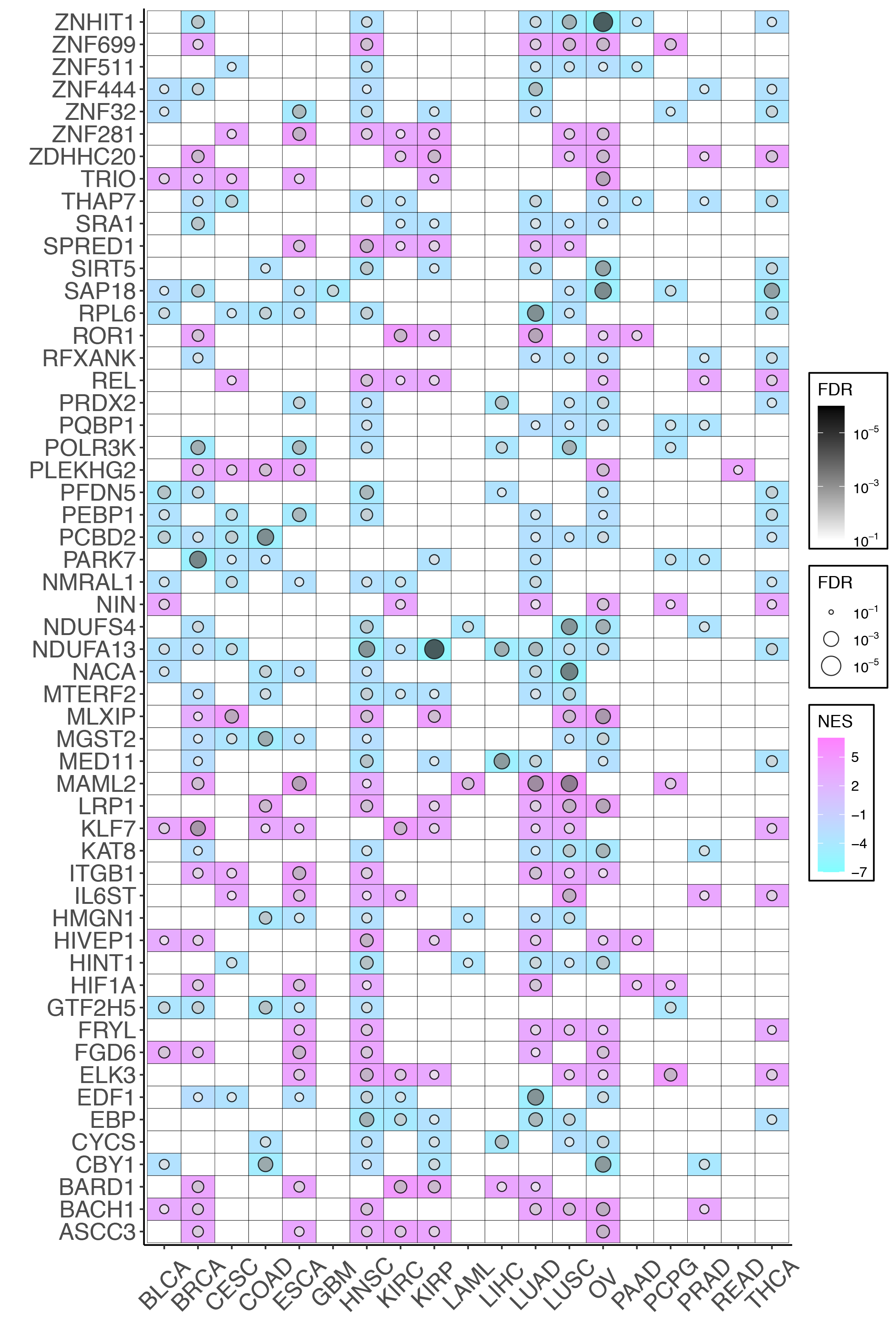

Supplement: Supplementary file 6 — Additional file 6: Fig. S6. Heatmap displaying the Normalized Enrichment Score (NES, shown as background color) and FDR (shown as dot size and color) of common TFs in different cancer types. Only TFs with an FDR < 0.05 are shown in the plot. [file 12967_2021_2889_MOESM6_ESM.png]

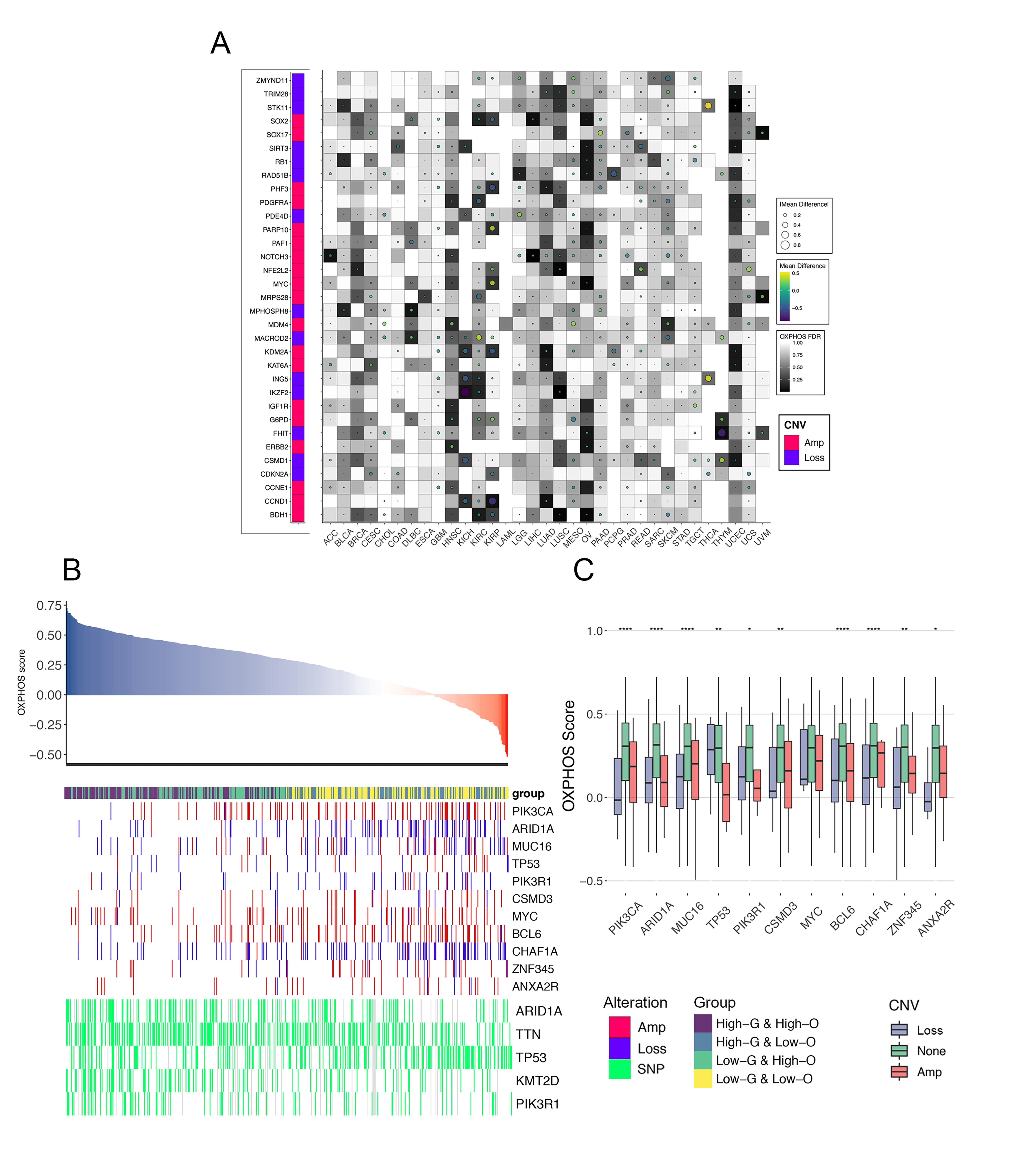

Supplement: Supplementary file 7 — Additional file 7: Fig. S7. (A) Association of OXPHOS score with copy number variation (CNVs) in oncogenes (labeled as red on the left) and tumor suppressor genes (labeled as blue on the left, Wilcoxon test). Dot size and color indicate the difference in mean OXPHOS between tumors with a CNV (gain for oncogene and loss for tumor suppressor gene) and those without. Background color indicates the FDR of Wilcoxon test. The empty region indicates that in that cancer type, there is no CNV of a specific gene. (B) Associations of SNVs and CNAs with OXPHOS score in UCEC patients. (C) Box plots displaying the distribution of OXPHOS score among patients with different CNV status (amplification, loss, and none) in UCEC. Within each group, the lines in the boxes represent the median value. The bottom and top of the boxes are the 25th and 75th percentiles (interquartile range). The whiskers encompass 1.5 times the interquartile range. The statistical difference of two scores was compared through the Wilcoxon test. ****, p < 0.0001. [file 12967_2021_2889_MOESM7_ESM.png]

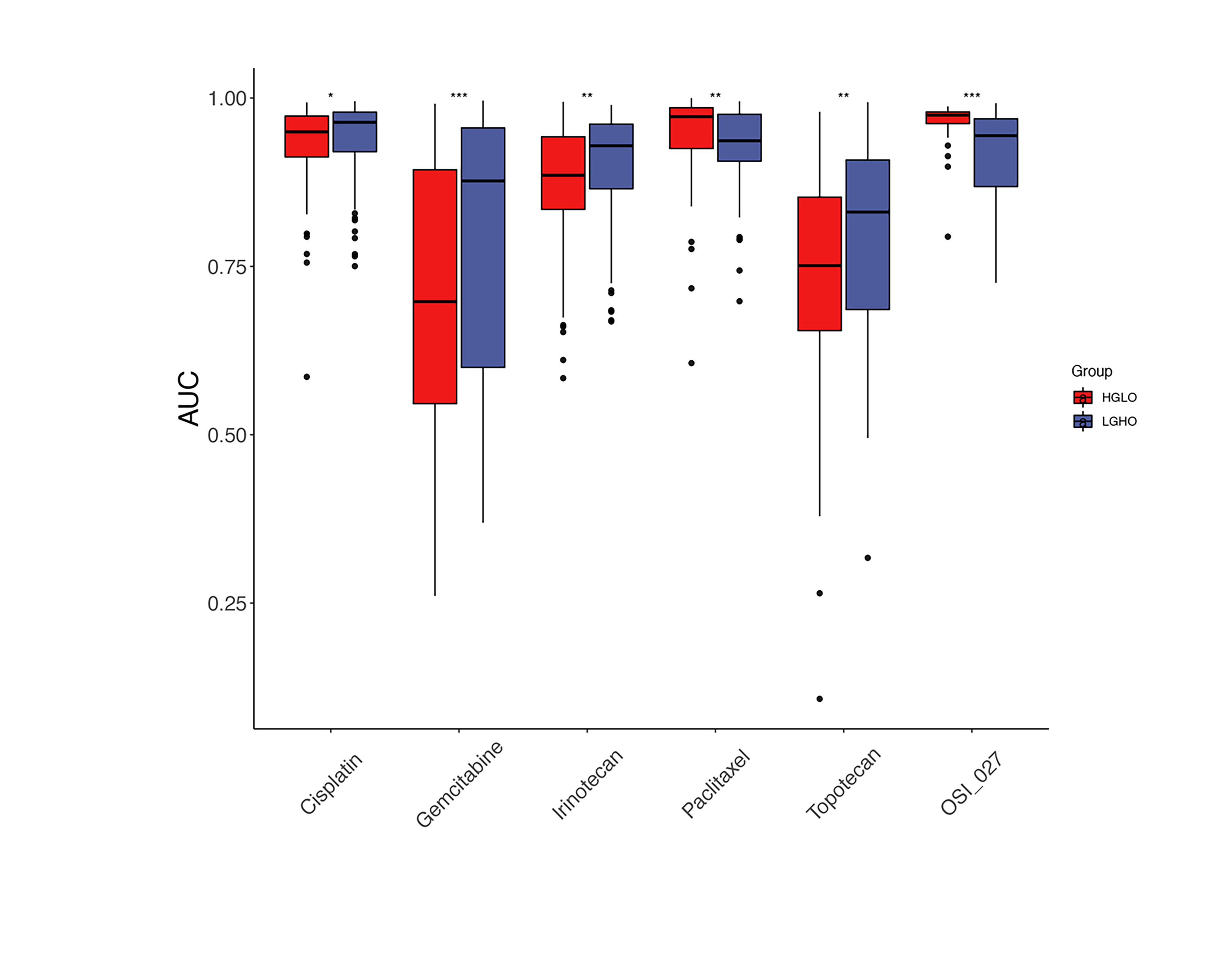

Supplement: Supplementary file 8 — Additional file 8: Fig. S8. Box plots displaying the distribution of AUC value, which reflects the sensitivity to specific anti-cancer drug, among cancer cells in different metabolic group. Within each group, the lines in the boxes represent the median value. The bottom and top of the boxes are the 25th and 75th percentiles (interquartile range). The whiskers encompass 1.5 times the interquartile range. The statistical difference of two scores was compared through the Wilcoxon test. *, p < 0.05; **, p < 0.01; ***, p < 0.001. [file 12967_2021_2889_MOESM8_ESM.png]
